# Tea saponin–soybean oil submicron emulsion promotes durable protective immunity against *Pasteurella multocida*

**DOI:** 10.3389/fimmu.2026.1899202

**Published:** 2026-07-20

**Authors:** Xuemei Cui, Le Yu, Ruiting Zhang, Yee Huang, Zizhe Hu, Xiaoyu Li, Quanan Ji, Tuanyuan Shi, Guolian Bao, Yan Liu

**Affiliations:** Institute of Animal Husbandry and Veterinary Science, Zhejiang Academy of Agricultural Sciences, Hangzhou, Zhejiang, China

**Keywords:** cellular immunity, durable immunity, lymph-node proteomics, Pasteurella multocida, protective immunity, submicron emulsion, tea saponin–soybean oil, vaccine adjuvant

## Abstract

**Background:**

Inactivated *Pasteurella multocida* (Pm) vaccines are safe and practical for veterinary use, but their protection is often limited by weak cellular immune activation and insufficient durability of antibody responses. This study investigated whether a tea saponin–soybean oil submicron emulsion could improve the durability, breadth and protective efficacy of immune responses induced by an inactivated *P. multocida* vaccine.

**Methods:**

Inactivated *P. multocida* antigen was formulated with tea saponin and soybean oil to generate TS-Pm. The formulation was characterized by particle size, polydispersity, surface charge, morphology and storage stability. Macrophage antigen uptake, cytokine secretion and activation-associated surface markers were examined *in vitro*. In mice, we evaluated antibody persistence, B-cell differentiation, splenic cellular immunity, pulmonary CD11b^+^ myeloid phenotypes, systemic tolerability, protection after lethal challenge and draining lymph-node proteomic profiles.

**Results:**

TS-Pm formed a stable oil-in-water submicron emulsion of approximately 300 nm with low polydispersity, negative surface charge and preserved morphology after 6 months at 4 °C. In RAW264.7 macrophages, TS-Pm increased antigen uptake, pro-inflammatory cytokine production and CD80, CD86 and MHC-II expression. Compared with alum-adjuvanted vaccine, TS-Pm maintained higher Pm-specific IgG responses, increased both IgG1 and IgG2a, and promoted plasmablast, plasma cell and germinal-center-associated B-cell responses. TS-Pm also enhanced antigen-responsive splenic proliferation, cytokine-producing CD4^+^ and CD8^+^ T-cell subsets, and activation-associated phenotypes in pulmonary CD11b^+^ myeloid cells. After lethal challenge, TS-Pm improved survival to 90%, compared with 60% for Alum-Pm, and reduced pulmonary bacterial burden and lung pathology without evidence of aggravated systemic injury. Draining lymph-node proteomics showed changes associated with antigen processing and presentation, NF-κB-related signaling and immune-cell trafficking.

**Conclusion:**

Tea saponin–soybean oil submicron emulsion improved the durability, breadth and protective efficacy of immune responses induced by an inactivated *P. multocida* vaccine. These findings identify TS-Pm as a practical plant-derived adjuvant formulation for inactivated bacterial vaccines requiring sustained humoral and cellular immunity.

## Introduction

*Pasteurella multocida* (Pm) is a Gram-negative opportunistic pathogen associated with respiratory and systemic infections across multiple host species and remains relevant to the One Health interface between animals and humans (1, 2). Infection by *P. multocida* often occurs within complex respiratory microbial communities, where bacterial pathogenicity is shaped by host susceptibility and interactions with other pathogens (3). Inactivated *P. multocida* vaccines are safe, manufacturable, and antigenically broad, but their protective efficacy is often constrained by weak cellular immune activation and limited durability of antibody responses (4, 5). Improving how inactivated bacterial antigens are organized and sensed by innate immune cells is therefore an important route to stronger and longer-lasting protection.

The performance of an inactivated vaccine is influenced not only by antigen composition, but also by the physical form in which the antigen is delivered. Emulsion structure, particle size, surface properties, and the mode of antigen association with the dispersed phase can affect uptake by innate immune cells and the subsequent adaptive response (6–8). Oil-in-water nanoemulsions and submicron emulsions are therefore more than passive dispersing systems. Their particulate structure can influence antigen exposure to phagocytic cells, macrophage activation, antibody production, cellular immunity, biocompatibility, and protection (8–10). For whole-cell bacterial antigens, which may aggregate after inactivation, a defined emulsion structure may be particularly useful for improving antigen accessibility.

Saponins are well suited to this formulation strategy because they can promote immune activation and antigen presentation, and recent advances in saponin adjuvant research have further reinforced their value as immune-active materials (11, 12). Particulate vaccine systems can also influence antigen access to lymphatic tissues and immune-cell compartments, thereby shaping the transition from innate recognition to adaptive priming (13, 14). Antigen processing and presentation then coordinate T-cell help, B-cell differentiation, and effector responses (15, 16). A submicron formulation combining a plant saponin with an oil phase could therefore provide both antigen organization and innate immune stimulation.

Tea saponin and soybean oil provide a practical plant-based combination for veterinary vaccine adjuvant design. Tea saponin is an amphiphilic plant saponin with immunomodulatory potential, whereas soybean oil provides an accessible and biocompatible oil phase. In veterinary vaccines, adjuvant selection must balance immune potency, safety, ease of preparation, component availability, and field use. Our previous studies showed that larger vegetable-oil emulsions enhanced immune responses to bacterial and viral antigens and were accompanied by molecular evidence of immune modulation, supporting further development of plant-oil-based adjuvants for different vaccine antigens (17, 18). The present study advances this line of work by reducing the tea saponin–soybean oil emulsion to a more uniform submicron size of approximately 300 nm. This refinement was intended to improve emulsion uniformity, storage stability, and antigen handling while retaining the practical advantages of readily available plant-derived components.

Here, we prepared a tea saponin–soybean oil submicron emulsion containing inactivated *P. multocida* antigen, designated TS-Pm, and examined whether it could improve the durability and breadth of vaccine-induced protective immunity. We first characterized the formulation and then evaluated macrophage antigen uptake and activation, antibody persistence, B-cell differentiation, T-cell responses, pulmonary myeloid phenotypes, systemic tolerability, and protection after bacterial challenge. Draining lymph-node proteomics was used to identify immune programs associated with TS-Pm immunization, including antigen processing and presentation, NF-κB-related signaling, and immune-cell trafficking. This design allowed us to link submicron formulation properties with innate immune activation, durable adaptive immunity, and protection against *P. multocida* challenge.

## Materials and methods

### Vaccine preparation and physicochemical characterization

*Pasteurella multocida* serotype A strain CVCC 500 was obtained from the China Veterinary Culture Collection Center. Bacteria were cultured in Martin broth at 37 °C for 18 h, harvested by centrifugation at 5, 000 × g for 15 min, washed three times with phosphate-buffered saline (PBS), and inactivated with 0.4% formalin at room temperature for 24 h. The inactivated bacterial suspension was resuspended in PBS and stored at 4 °C until use. Complete inactivation was confirmed by plating aliquots on Martin broth agar and verifying the absence of bacterial growth after incubation at 37 °C for 48 h.

The tea saponin–soybean oil submicron emulsion containing inactivated *P. multocida* antigen was designated TS-Pm. To prepare TS-Pm, the oil and aqueous phases were mixed at room temperature with Span 80 and Tween 80 at final concentrations of 7% and 4%, respectively, at an oil-to-aqueous phase volume ratio of 2:1. The mixture was first homogenized using an IKA T10 ULTRA-TURRAX homogenizer (12, 000 rpm, 1 min; Staufen, Germany) to form a coarse emulsion, followed by high-pressure homogenization using an AH-1500 homogenizer (ATS Engineering, Canada) at 1000 bar for 8 cycles. The resulting submicron emulsion was prepared under aseptic conditions and stored at 4 °C. Alum-Pm was prepared by adsorbing inactivated antigen onto aluminum hydroxide gel (Thermo Fisher, Massachusetts, USA) at a 1:2 volume ratio with stirring for 20–30 min. TS-Pm, Alum-Pm, and Pm antigen alone contained inactivated *P. multocida* standardized to 5 × 10^8^ CFU equivalents/mL.

Hydrodynamic diameter, polydispersity index (PDI), and zeta potential were measured by dynamic light scattering using a Zetasizer Nano ZS (Malvern Instruments, UK). Samples were diluted 1:10 in deionized water before measurement, and each measurement was performed in triplicate. Particle morphology was examined by transmission electron microscopy (TEM; JEM-2100, JEOL, Japan). Briefly, 10 μL of diluted sample was placed on carbon-coated copper grids, negatively stained with 2% phosphotungstic acid, air-dried, and imaged at 100 kV. To assess storage stability, TS-Pm was stored at 4 °C for 6 months and then re-examined by dynamic light scattering and TEM for size distribution, PDI, zeta potential, and morphology.

### *In vitro* macrophage assays

#### Cell culture and CCK-8 assay

RAW264.7 cells (ATCC TIB-71, Manassas, VA, USA) were cultured in Dulbecco’s modified Eagle medium (DMEM; Gibco, USA) supplemented with 10% fetal bovine serum (Gibco), 100 U/mL penicillin, and 100 μg/mL streptomycin. Cells were maintained at 37 °C in a humidified incubator with 5% CO_2_.

For CCK-8 assays, RAW264.7 cells were seeded in 96-well plates at 5 × 10³ cells per well. Cells were treated with 100 μL of inactivated *P. multocida* antigen at 5 × 10^7^, 10^6^, 10^5^, or 10^4^ CFU equivalents/mL combined with tea saponin at 2000, 200, 20, or 2 ng/mL, with or without soybean oil. Lipopolysaccharide (LPS; 10 μg/mL; Sigma, USA) was used as a positive control, and untreated cells served as baseline controls. After 48 h, CCK-8 reagent (Dojindo, Japan) was added, and absorbance at 450 nm was measured to estimate cell metabolic activity.

#### Macrophage cytokine measurement

RAW264.7 cells were seeded in 24-well plates at 1 × 10^5^ cells per well and treated with tea saponin (200 ng/mL), Pm antigen (5 × 10^6^ CFU equivalents/mL), TS-Pm at a dilution corresponding to 200 ng/mL tea saponin and 5 × 10^6^ CFU equivalents/mL antigen, or LPS for 24 h. Culture supernatants were collected, and TNF-α, IL-1β, IL-6, and IL-12p70 were measured using ELISA kits (MultiSciences, Hangzhou, China) according to the manufacturer’s instructions.

#### Antigen uptake assay

FITC-conjugated ovalbumin (FITC-OVA; Sigma, USA) was used as a fluorescent model antigen to assess antigen uptake. RAW264.7 cells were treated with the indicated formulations and incubated with FITC-OVA at 20 μg/mL for 4 h. Cells were washed with PBS, harvested, and analyzed by flow cytometry using a BD FACSCanto II cytometer (BD Biosciences, USA). Data were analyzed using FlowJo v10 software (TreeStar, USA).

#### Flow cytometry for macrophage surface markers

After 24 h of treatment, RAW264.7 cells were collected and blocked with anti-CD16/32 antibody (BioLegend, USA). Cells were then stained with fluorochrome-conjugated antibodies against CD80, CD86, CD206, and MHC-II (BioLegend, USA). Samples were acquired on a BD FACSCanto II cytometer, with at least 10, 000 events analyzed per sample. The CD80/CD206 ratio was calculated to summarize the balance of activation-associated surface phenotypes.

#### Animals and ethics statement

Specific pathogen-free female ICR mice aged 6–8 weeks were obtained from the Shanghai Animal Center, China. Mice were housed under standard conditions at 22 ± 3 °C and 50 ± 5% relative humidity, with a 12-h light/dark cycle and free access to food and water. Animals were acclimated for at least 7 days before experimentation and were randomly assigned to treatment groups.

All mouse experiments, including immunization, challenge, and sample collection, were approved by the Animal Welfare and Ethics Committee of the Zhejiang Academy of Agricultural Sciences, Hangzhou, China (approval no. ZAASLA2024092001). All procedures were conducted in accordance with institutional and national guidelines for the care and use of laboratory animals. For blood sampling and terminal tissue collection, mice were anesthetized by intraperitoneal injection of sodium pentobarbital at 50 mg/kg. Blood samples were collected from the retro-orbital venous plexus under anesthesia. At the indicated experimental endpoints, mice were euthanized by cervical dislocation under anesthesia, and death was confirmed before tissue collection.

#### Mouse immunization protocols

Mice were immunized intramuscularly with 100 μL of the indicated formulations on days 0 and 14. For the formulation screening experiment, mice were assigned to Saline, Pm, SO+Pm, TS (2, 4, or 6 μg)+Pm, or SO+TS (2, 4, or 6 μg)+Pm groups. The group allocation, formulation composition, antigen/adjuvant dose, injection volume, and immunization schedule are summarized in **Table 1**. Serum samples were collected at the indicated time points to measure antigen-specific antibody responses. The 4 μg SO+TS+Pm formulation induced the strongest antibody response and was designated TS-Pm for subsequent experiments.

**Table 1:** Group allocation and immunization schedule for the formulation screening experiment.

Group	Formulation per dose	Injection volume	Immunization schedule
Saline	Saline	100 μL	Days 0 and 14
Pm	Inactivated *P. multocida* antigen, 5 × 10^7^ CFU equivalents per dose	100 μL	Days 0 and 14
SO+Pm	Soybean oil plus inactivated *P. multocida* antigen, 5 × 10^7^ CFU equivalents per dose	100 μL	Days 0 and 14
TS(2 μg)+Pm	Tea saponin, 2 μg, plus inactivated *P. multocida* antigen, 5 × 10^7^ CFU equivalents per dose	100 μL	Days 0 and 14
TS(4 μg)+Pm	Tea saponin, 4 μg, plus inactivated *P. multocida* antigen, 5 × 10^7^ CFU equivalents per dose	100 μL	Days 0 and 14
TS(6 μg)+Pm	Tea saponin, 6 μg, plus inactivated *P. multocida* antigen, 5 × 10^7^ CFU equivalents per dose	100 μL	Days 0 and 14
SO+TS(2 μg)+Pm	Soybean oil plus tea saponin, 2 μg, and inactivated *P. multocida* antigen, 5 × 10^7^ CFU equivalents per dose	100 μL	Days 0 and 14
SO+TS(4 μg)+Pm	Soybean oil plus tea saponin, 4 μg, and inactivated *P. multocida* antigen, 5 × 10^7^ CFU equivalents per dose	100 μL	Days 0 and 14
SO+TS(6 μg)+Pm	Soybean oil plus tea saponin, 6 μg, and inactivated *P. multocida* antigen, 5 × 10^7^ CFU equivalents per dose	100 μL	Days 0 and 14

SO, soybean oil; TS, tea saponin; Pm, inactivated *Pasteurella multocida* antigen. The inactivated Pm antigen was standardized to 5 × 10^8^ CFU equivalents/mL, corresponding to 5 × 10^7^ CFU equivalents per 100 μL dose. SO+TS(4 μg)+Pm was designated TS-Pm for subsequent experiments.

For the antibody persistence experiment, mice were immunized with Saline, Pm, Alum-Pm, or TS-Pm. Blood samples were collected from day 7 to day 42 after booster immunization to evaluate the kinetics of Pm-specific IgG responses. Spleens were collected at day 42 after booster immunization for B-cell subset analysis.

For analysis of splenic cellular immunity and pulmonary myeloid cell phenotypes, mice were immunized with Saline, Pm, Alum-Pm, or TS-Pm. Spleens and lungs were collected at day 21 after booster immunization. Serum samples were stored at −80 °C until analysis.

### Enzyme-linked immunosorbent assay

Pm-specific total IgG, IgG1, and IgG2a were measured by indirect ELISA. Ninety-six-well ELISA plates were coated overnight at 4 °C with 100 μL per well of inactivated *P. multocida* antigen at 1 μg/mL in carbonate-bicarbonate buffer, pH 9.6. Plates were washed three times with PBS containing 0.05% Tween 20 and blocked with 5% skim milk in PBS for 2 h at 37 °C. Diluted serum samples (1:2000) were added in triplicate and incubated at 37 °C for 1 h. After washing, HRP-conjugated goat anti-mouse IgG, IgG1, or IgG2a antibodies were added at 1:10, 000 and incubated for 1 h at 37 °C. Plates were developed with TMB substrate for 15 min in the dark, and the reaction was stopped with 2 M H_2_SO_4_. Absorbance was measured at 450 nm using a Thermo Multiskan microplate reader (Thermo Fisher, Shanghai, China).

### Splenocyte proliferation assay

At day 21 after booster immunization, spleens were collected aseptically from immunized mice. Single-cell suspensions were prepared by mechanical disruption and filtration through 70-μm strainers, followed by red blood cell lysis with ACK lysis buffer. Splenocytes were plated in 96-well plates at 5 × 10^6^ cells/mL and stimulated with inactivated *P. multocida* antigen at 1 × 10^9^ CFU equivalents/mL, LPS at 10 μg/mL (Sigma, USA), or concanavalin A (ConA) at 5 μg/mL (Sigma, USA). After 72 h, proliferative responses were estimated using a CCK-8 assay, and absorbance at 450 nm was measured using a BioTek Synergy H1 reader (Agilent, USA).

### Cytokine measurement in serum and splenocyte cultures

Blood samples were collected at day 21 after booster immunization, and sera were isolated by centrifugation at 3, 000 × g for 10 min. Spleens were collected at the same time point, and splenocytes were prepared as described above. Cells were plated in 96-well plates at 5 × 10^6^ cells/mL and restimulated with inactivated *P. multocida* antigen at 10 μg/mL for 72 h. Culture supernatants were harvested. IFN-γ, IL-4, IL-5, and IL-17 concentrations in serum and splenocyte culture supernatants were measured using ELISA kits (MultiSciences, China) according to the manufacturer’s instructions.

### Quantitative real-time PCR

Total RNA was extracted from splenocytes or draining lymph nodes using TRIzol reagent (Invitrogen, USA). cDNA was synthesized using the PrimeScript RT reagent kit (Takara, Japan) according to the manufacturer’s instructions. Quantitative PCR was performed with SYBR Green Master Mix (Applied Biosystems, USA) on a QuantStudio 6 Flex system (Thermo Fisher, USA).

For splenocyte samples, *STAT4*, *STAT6*, and *RORγt* expression was measured. For draining lymph node samples, selected targets identified from the proteomic analysis, including Nlrc4, Traf6, Tap1, Tap2, CD40, and Cxcr3, were analyzed. GAPDH was used as the reference gene. Relative expression levels were calculated using the 2−ΔΔCt method. Primer sequences are listed in **Supplementary Table 1**.

### Flow cytometry analysis

#### Splenic B-cell subsets

Spleens were collected at day 42 after booster immunization from mice immunized with Saline, Pm, Alum-Pm, or TS-Pm. Single-cell suspensions were prepared by mechanical disruption and filtration through 70-μm strainers, followed by red blood cell lysis. Splenocytes were stained on ice with fluorochrome-conjugated antibodies against CD19, CD138, CD38, and Fas (BioLegend, USA). Plasmablasts were defined as CD19^+^CD138^−^CD38^+^ cells, plasma cells as CD19^+^CD138^+^CD38^+^ cells, and germinal center B cells as CD19^+^CD38^+^Fas^+^ cells. These subsets were quantified as percentages of total CD19^+^ B cells.

#### Splenic T-cell subsets and intracellular cytokine staining

Spleens were collected at day 21 after booster immunization from mice immunized with Saline, Pm, Alum-Pm, or TS-Pm. Splenocytes were blocked with anti-CD16/32 antibody and stained with antibodies against CD3, CD4, and CD8 (BioLegend, USA). For intracellular cytokine staining, cells were analyzed directly ex vivo without antigen restimulation. After fixation and permeabilization using a Cytofix/Cytoperm kit (BD Biosciences, USA), cells were stained intracellularly with antibodies against IFN-γ, IL-4, and IL-17 (BioLegend, USA). CD4^+^IFN-γ^+^, CD8^+^IFN-γ^+^, CD4^+^IL-4^+^, and CD4^+^IL-17^+^ subsets were quantified within the CD3^+^ T-cell population.

#### Pulmonary CD11b^+^ myeloid cells

Lungs were collected at day 21 after booster immunization from mice immunized with Saline, Pm, Alum-Pm, or TS-Pm. Lung tissues were minced and digested with 1 mg/mL collagenase IV (Solarbio, China) and 50 μg/mL DNase I (Sigma, USA) for 30 min at 37 °C. Digests were filtered through 70-μm strainers and treated with ACK lysis buffer to remove erythrocytes. Lung leukocytes were blocked with anti-CD16/32 antibody and stained with antibodies against CD11b, CD80, CD86, MHC-I, and MHC-II (BioLegend, USA). 7-AAD was used to exclude nonviable cells.

All flow cytometry data were acquired on a BD FACSCanto II cytometer and analyzed using FlowJo v10.8.1 software. The corresponding gating strategies are provided in the Supplementary Information.

### Challenge experiment and bacterial load determination

At day 42 after booster immunization, mice were challenged intravenously with 0.1 mL of a virulent *P. multocida* suspension containing 1 × 10² CFU per mouse. Survival was monitored daily for 10 days. For terminal pulmonary bacterial burden analysis, lungs were collected from mice that died during the 10-day post-challenge observation period or from surviving mice euthanized at the scheduled day-10 endpoint. The lungs were collected aseptically, snap-frozen in liquid nitrogen, rapidly transferred to −80 °C storage, and processed together for bacterial enumeration. Lung tissues were homogenized in 1 mL sterile PBS, serially diluted tenfold, and plated in duplicate on tryptic soy agar (Oxoid, UK). After incubation at 37 °C for 20 h, colonies were counted, and pulmonary bacterial loads were calculated as CFU per lung. Preliminary intranasal challenge attempts did not yield consistent mortality across a practicable dose range. Therefore, intravenous challenge was used as a stringent and reproducible severe disease model, and pulmonary bacterial burden and lung pathology were assessed after challenge.

### Histopathology and lung pathology scoring

Major organs, including heart, liver, spleen, lung, and kidney, were collected at day 10 after challenge, fixed in 4% neutral buffered paraformaldehyde for 24 h, embedded in paraffin, sectioned at 5 μm, and stained with hematoxylin and eosin. Representative pathological features were documented by light microscopy.

Lung pathology was evaluated using a semiquantitative scoring system based on inflammatory cell infiltration, alveolar septal thickening or edema, and hemorrhage or congestion. Each parameter was graded on a 0–4 scale and summed for statistical analysis. Lung sections from five mice per group were scored by an investigator blinded to group allocation using multiple nonoverlapping fields per section.

### Confocal immunofluorescence of lung sections

Lung lobes collected at day 10 after challenge were fixed in 4% paraformaldehyde, cryoprotected in 30% sucrose, embedded in OCT compound, and cryosectioned at 8 μm. Sections were permeabilized with 0.3% Triton X-100 and blocked with 5% bovine serum albumin. Slides were incubated overnight at 4 °C with primary antibodies against F4/80, CD86, CD206, MHC-I, MHC-II, IL-4, IL-10, and IL-17 (Abcam and BioLegend). After washing, sections were incubated with species-appropriate Alexa Fluor-conjugated secondary antibodies (Invitrogen, USA) for 1 h at room temperature. Nuclei were counterstained with DAPI (Beyotime, China). Images were acquired using a Zeiss LSM 880 confocal laser scanning microscope and processed with ZEN software.

### Hematology

Peripheral blood was collected from the retro-orbital venous plexus at days 7, 21, and 42 after booster immunization and at day 45, corresponding to 3 days after challenge. Complete blood counts were performed using an automated hematology analyzer (Mindray BC-5000Vet, China). Total white blood cells, lymphocytes, neutrophils, and monocytes were recorded.

### Antioxidant assays

Serum samples collected at day 21 after booster immunization and day 45, corresponding to 3 days after challenge, were analyzed for antioxidant-related biomarkers. Total antioxidant capacity, superoxide dismutase activity, glutathione peroxidase activity, and malondialdehyde levels were measured using commercial assay kits (Nanjing Jiancheng Bioengineering Institute, China) according to the manufacturer’s instructions.

### Biochemical assays

Serum samples collected at day 3 after prime immunization and day 45, corresponding to 3 days after challenge, were subjected to clinical chemistry analysis. Alanine aminotransferase, aspartate aminotransferase, albumin, total protein, creatinine, and creatine kinase were measured using an automated biochemical analyzer and corresponding reagent kits. These parameters were used to evaluate hepatic, renal, and muscular function after immunization and challenge.

### Draining lymph-node proteomic analysis

Draining lymph nodes were collected at day 21 after booster immunization (n = 3 per group), snap-frozen, and homogenized. Proteins were extracted in lysis buffer containing protease inhibitors and quantified using a BCA assay. Equal amounts of protein from each sample were reduced, alkylated, and digested with trypsin. Peptides were analyzed by LC-MS/MS on a Q Exactive Plus Orbitrap mass spectrometer coupled to an Easy-nLC 1200 system. Raw data were processed using MaxQuant v1.6.10.43 against the UniProt *Mus musculus* database.

Differentially expressed proteins were defined using a fold change ≥1.5 or ≤0.67 and P < 0.05. Gene Ontology and KEGG enrichment analyses were performed in R using clusterProfiler v4.2.0 with annotation support from DAVID. Gene set enrichment analysis was conducted using Broad Institute software v4.3.2, with FDR < 0.25 considered significant. Protein interaction networks were constructed using STRING v11.5 and visualized in Cytoscape v3.9.0. Interactions were displayed across STRING combined score confidence levels from 0.5 to 0.9. Node size was scaled to degree centrality, and hub nodes were prioritized by degree and betweenness centrality where indicated. The proteomics data are available via ProteomeXchange under accession number PXD071626.

### Statistical analysis

Statistical analyses were performed using GraphPad Prism 8.0. One-way ANOVA with Tukey’s multiple comparisons test was used for comparisons among more than two groups under a single condition. Two-way ANOVA with Sidak’s multiple comparisons test was used for time-course and multifactorial experiments. Survival curves were compared using the log-rank test. Data are presented as mean ± SEM, and P < 0.05 was considered statistically significant.

## Results

### TS-Pm forms a defined and stable submicron emulsion

**Figure 1:**
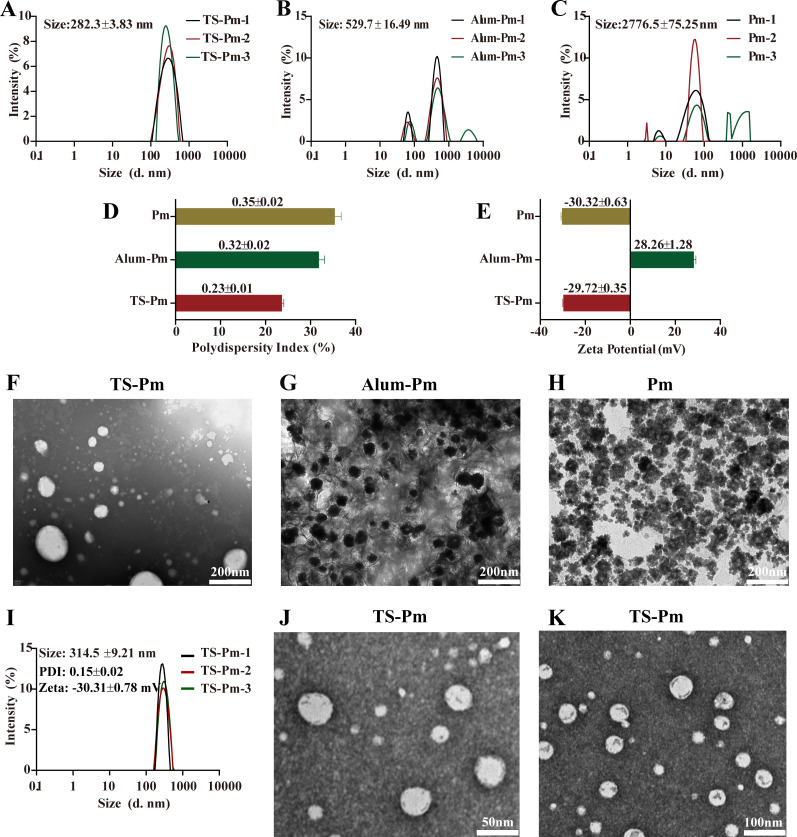
Physicochemical characterization and storage stability of TS-Pm. **(A–C)** Particle size distributions of TS-Pm, Alum-Pm and Pm measured by dynamic light scattering. **(D)** Polydispersity index. **(E)** Zeta potential. **(F–H)** Transmission electron microscopy images of TS-Pm, Alum-Pm and Pm. Scale bars, 200 nm. **(I)** Hydrodynamic diameter, polydispersity index and zeta potential of TS-Pm after storage at 4 °C for 6 months. **(J, K)** Transmission electron microscopy images of TS-Pm after 6 months of storage. Scale bars, 50 nm in J and 100 nm in K. Data are presented as mean ± SEM (n = 3).

Dynamic light scattering showed that TS-Pm had a mean hydrodynamic diameter of 282.3 ± 3.83 nm and a polydispersity index (PDI) of 0.23 ± 0.01 (**Figures 1A, D**). Alum-Pm had a larger particle size of 529.7 ± 16.49 nm and a PDI of 0.32 ± 0.02, whereas free Pm antigen formed aggregates larger than 2700 nm with a PDI of 0.35 ± 0.02 (**Figures 1B–D**). The zeta potential of TS-Pm was −29.7 ± 0.4 mV, close to that of free Pm antigen, while Alum-Pm carried a positive surface charge (**Figure 1E**). TEM imaging showed approximately spherical and well-dispersed TS-Pm particles, in contrast to the irregular clusters formed by Alum-Pm and the dense aggregates observed for free Pm antigen (**Figures 1F–H**).

The storage stability of TS-Pm was next examined at 4 °C. After 6 months, TS-Pm retained a submicron size, with a mean diameter of 314.5 ± 9.21 nm, a PDI of 0.15 ± 0.02, and a zeta potential of −30.4 ± 0.8 mV (**Figure 1I**). TEM imaging after storage showed preserved spherical morphology without obvious aggregation (**Figures 1J, K**). Together, the particle size, surface charge, morphology, and storage profile defined TS-Pm as a stable oil-in-water submicron emulsion for immunological evaluation.

### TS-Pm enhances antigen uptake and macrophage activation *in vitro*

**Figure 2:**
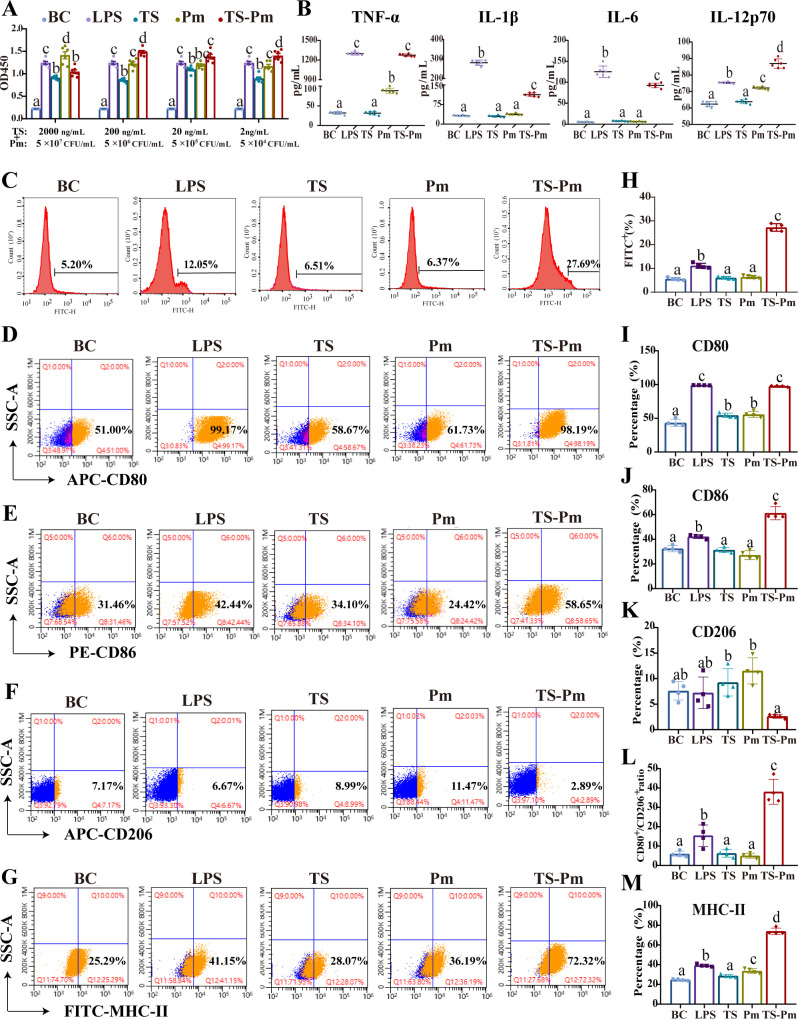
TS-Pm enhances antigen uptake and activation in macrophages. **(A)** Metabolic activity of RAW264.7 cells after treatment with inactivated Pm antigen with or without tea saponin (TS) and/or soybean oil (SO) at the indicated concentrations. **(B)** TNF-α, IL-1β, IL-6 and IL-12p70 concentrations in culture supernatants after 24 h of stimulation. **(C, H)** Representative flow cytometry histograms and quantification of FITC-OVA uptake. **(D–G)** Representative flow cytometry plots showing CD80, CD86, CD206 and MHC-II expression on RAW264.7 cells. **(I–M)** Quantification of surface marker expression and the CD80/CD206 ratio. Data are presented as mean ± SEM (n = 4). Statistical significance was determined by one-way ANOVA followed by Tukey’s multiple comparisons test. Groups not sharing a letter differ significantly (P < 0.05). Gating strategies are shown in **Supplementary Figure 1**.

RAW264.7 macrophages were used to assess the early innate cellular activity of TS-Pm. In the CCK-8 assay, cellular metabolic activity varied according to the concentrations of inactivated Pm antigen and tea saponin. The combination of 5 × 10^6^ CFU equivalents/mL Pm antigen and 200 ng/mL tea saponin produced the highest signal among the TS-Pm conditions tested and exceeded the corresponding Pm antigen or tea saponin alone groups (**Figure 2A**). Across low and intermediate concentration ranges, the combined formulation generally produced stronger metabolic activity than either component alone, whereas the highest antigen and tea saponin dose did not further increase the response.

TS-Pm increased macrophage cytokine production. TNF-α, IL-1β, IL-6, and IL-12p70 levels were higher after TS-Pm treatment than after treatment with Pm antigen or tea saponin alone, with IL-12p70 showing a prominent increase in the TS-Pm group (**Figure 2B**). In the FITC-OVA uptake assay, TS-Pm increased the proportion of FITC-positive RAW264.7 cells compared with Pm antigen, tea saponin, or LPS treatment (**Figures 2C, H**). Flow cytometry further showed increased CD80, CD86, and MHC-II expression in TS-Pm-treated macrophages, together with reduced CD206 expression and a higher CD80/CD206 ratio (**Figures 2D–G, I–M**). The gating strategies are shown in **Supplementary Figure 1**. These uptake, cytokine, and surface-marker profiles support enhanced antigen internalization and activation-associated macrophage polarization after TS-Pm treatment.

### TS-Pm sustains antibody responses and promotes B-cell differentiation

**Figure 3:**
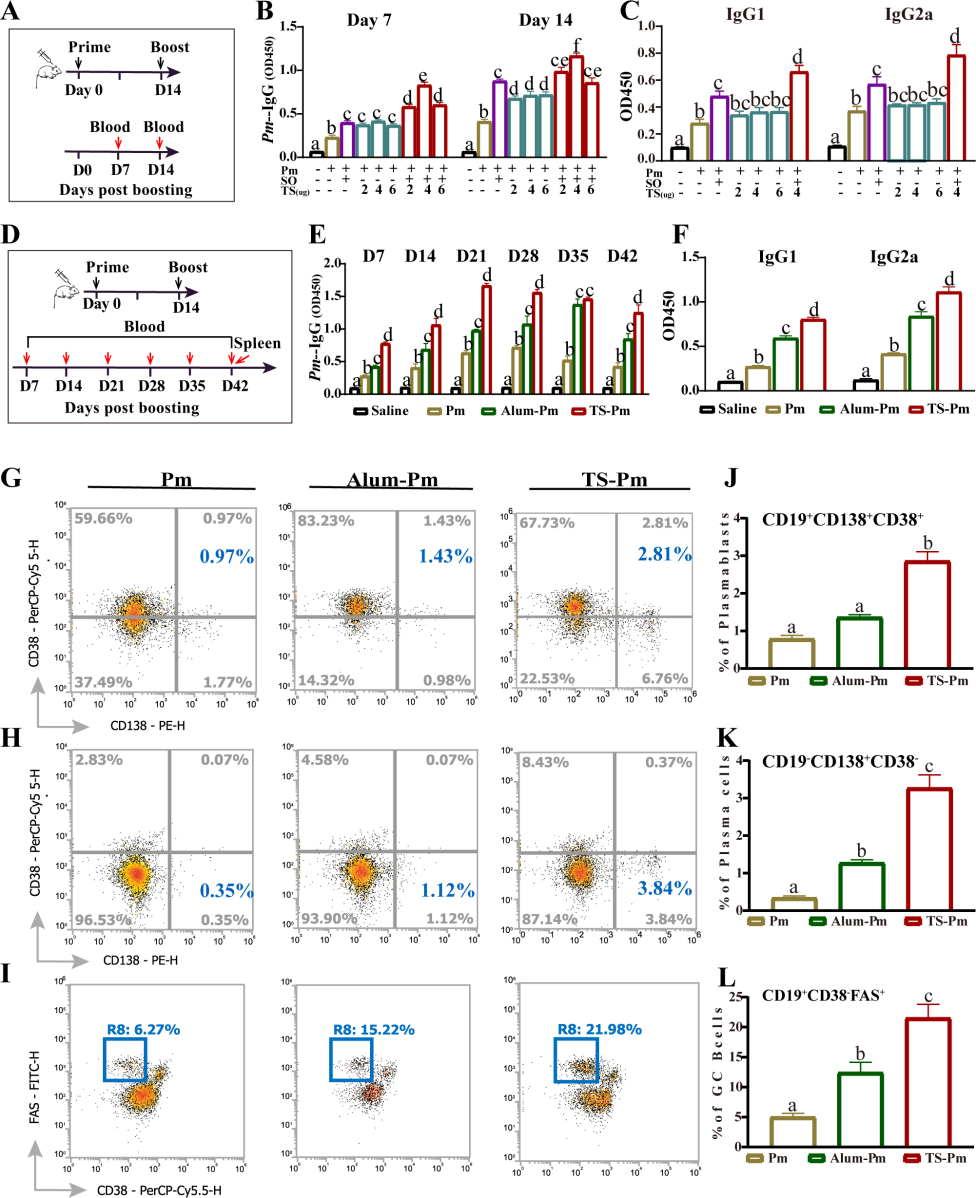
TS-Pm promotes durable antibody responses and B cell differentiation. **(A)** Immunization schedule for formulation screening. ICR mice were immunized intramuscularly on days 0 and 14 with saline, Pm, SO+Pm, TS (2, 4 or 6 μg)+Pm, or SO+TS (2, 4 or 6 μg)+Pm. **(B)** Serum anti-Pm IgG titers at days 7 and 14 after booster immunization. **(C)** Serum IgG1 and IgG2a subclasses at day 14 after booster immunization. The 4 μg SO+TS+Pm formulation induced the strongest antibody responses and was designated TS-Pm. **(D)** Immunization schedule for the antibody persistence experiment. **(E)** Serum anti-Pm IgG titers from day 7 to day 42 after booster immunization. **(F)** Serum IgG1 and IgG2a subclasses at day 21 after booster immunization. **(G–I)** Representative flow cytometry plots of splenic plasmablasts (CD19^+^CD138^−^CD38^+^), plasma cells (CD19^+^CD138^+^CD38^+^) and germinal center B cells (CD19^+^CD38^+^Fas^+^) at day 42 after booster immunization. **(J–L)** Quantification of the corresponding B cell populations. Data are presented as mean ± SEM. For serum antibody analyses, n = 6 mice per group. For flow cytometry, n = 4 mice per group. Statistical significance was determined by one-way ANOVA followed by Tukey’s multiple comparisons test, except for antibody kinetics in E, which were analyzed by two-way ANOVA followed by Sidak’s multiple comparisons test. Groups not sharing a letter differ significantly (P < 0.05). The gating strategy is shown in **Supplementary Figure 2**.

The contribution of tea saponin and soybean oil to humoral immunity was first examined in a formulation screening experiment (**Figure 3A**). Tea saponin alone induced only modest increases in anti-Pm IgG compared with Pm antigen alone. Incorporation of soybean oil further increased the antibody response, and the SO+TS+Pm formulation containing 4 μg tea saponin produced the strongest IgG response among the combinations tested (**Figure 3B**). This formulation was designated TS-Pm for subsequent experiments. At day 14 after booster immunization, TS-Pm increased both IgG1 and IgG2a responses, indicating a broader antibody subclass response than Pm antigen alone (**Figure 3C**).

Antibody persistence was then compared among the Saline, Pm, Alum-Pm, and TS-Pm groups (**Figure 3D**). From day 7 to day 42 after booster immunization, TS-Pm maintained higher Pm-specific IgG levels than Alum-Pm or Pm at each measured time point, with the highest response observed around day 21 (**Figure 3E**). At this peak response, TS-Pm also induced higher IgG1 and IgG2a levels than Alum-Pm (**Figure 3F**). Flow cytometry at day 42 showed increased splenic plasmablasts, plasma cells, and germinal center B cells in the TS-Pm group compared with the Alum-Pm and Pm groups (**Figures 3G–L**). The gating strategy for B-cell subset analysis is shown in **Supplementary Figure 2**. Thus, the tea saponin–soybean oil emulsion increased antibody magnitude, extended antibody persistence, and promoted B-cell differentiation.

### TS-Pm broadens splenic cellular immune responses

**Figure 4:**
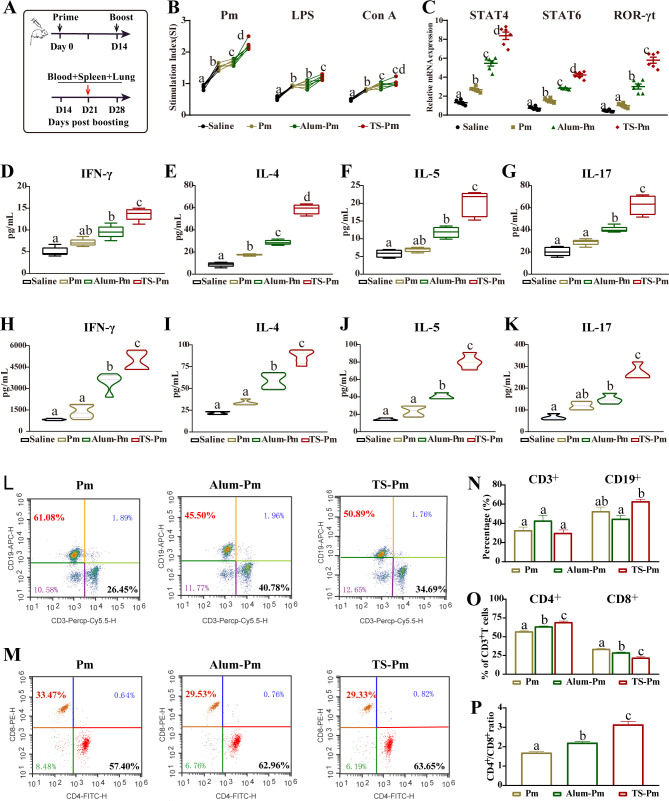
TS-Pm enhances splenic cellular immune responses. **(A)** Immunization schedule. **(B)** Ex vivo splenocyte proliferation after restimulation with inactivated Pm antigen, LPS or ConA. **(C)** Relative mRNA expression of *STAT4*, *STAT6* and *RORγt* normalized to GAPDH. **(D–G)** Serum cytokine concentrations. **(H–K)** Cytokine concentrations in splenocyte culture supernatants after antigen restimulation. **(L, M)** Representative flow cytometry plots of CD3^+^ T cells, CD19^+^ B cells, and CD4^+^ and CD8^+^ T cell subsets. **(N–P)** Quantification of CD3^+^, CD19^+^, CD4^+^ and CD8^+^ cells and the CD4^+^/CD8^+^ ratio. Data are presented as mean ± SEM (n = 6 mice per group). Statistical significance was determined by one-way ANOVA followed by Tukey’s multiple comparisons test. Groups not sharing a letter differ significantly (P < 0.05). The gating strategy is shown in **Supplementary Figure 1**.

Splenic immune responses were evaluated at day 21 after booster immunization (**Figure 4A**). Splenocytes from TS-Pm-immunized mice showed stronger proliferation after restimulation with Pm antigen or LPS than splenocytes from the Alum-Pm and Pm groups (**Figure 4B**). Under ConA stimulation, both TS-Pm and Alum-Pm induced stronger proliferation than Pm, with no clear difference between the two adjuvanted groups.

TS-Pm increased *STAT4*, *STAT6*, and *RORγt* expression in splenocytes compared with Alum-Pm and Pm (**Figure 4C**). Consistent with these transcriptional changes, TS-Pm increased IFN-γ, IL-4, IL-5, and IL-17 levels in serum and in antigen-restimulated splenocyte supernatants, whereas Alum-Pm generally produced intermediate responses (**Figures 4D–K**). Flow cytometry showed comparable total CD3^+^ T-cell frequencies across groups, while CD19^+^ B-cell frequencies were higher in the TS-Pm and Alum-Pm groups than in the Pm group (**Figures 4L–P**). The CD4^+^/CD8^+^ ratio was also higher in the TS-Pm group than in the Alum-Pm and Pm groups. The corresponding gating strategy is provided in **Supplementary Figure 1**. These results indicate that TS-Pm enhanced antigen-responsive splenic proliferation and broadened cytokine production after immunization.

### TS-Pm increases cytokine-producing T cells and pulmonary myeloid activation

**Figure 5:**
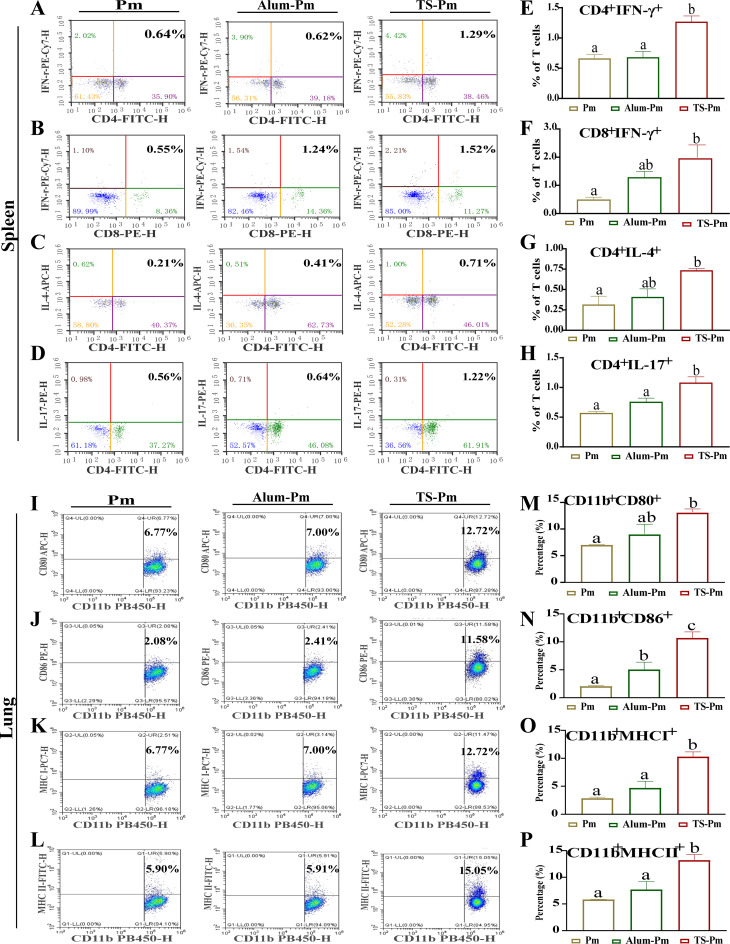
TS-Pm increases cytokine producing T cells and pulmonary CD11b^+^ myeloid activation. **(A–D)** Representative flow cytometry plots of CD4^+^IFN-γ^+^, CD8^+^IFN-γ^+^, CD4^+^IL-4^+^ and CD4^+^IL-17^+^ T cells. **(E–H)** Quantification of the corresponding cytokine positive T cell subsets. **(I–L)** Representative flow cytometry plots of CD80, CD86, MHC-I and MHC-II expression on lung CD11b^+^ myeloid cells. **(M–P)** Quantification of CD80, CD86, MHC-I and MHC-II expression on lung CD11b^+^ myeloid cells. Data are presented as mean ± SEM (n = 6 mice per group). Statistical significance was determined by one-way ANOVA followed by Tukey’s multiple comparisons test. Groups not sharing a letter differ significantly (P < 0.05). Gating strategies are shown in **Supplementary Figure 1**.

Intracellular cytokine staining was used to further define T-cell responses after immunization. TS-Pm increased the proportions of CD4^+^IFN-γ^+^ and CD4^+^IL-17^+^ T cells compared with Alum-Pm or Pm (**Figures 5A–H**). TS-Pm also increased CD8^+^IFN-γ^+^ and CD4^+^IL-4^+^ T-cell subsets relative to Pm, whereas Alum-Pm showed smaller increases.

Pulmonary CD11b^+^ myeloid cells were analyzed to assess activation-associated phenotypes in a major target organ of *P. multocida* infection. Within the CD11b^+^ gate, TS-Pm immunization was associated with higher frequencies of CD86^+^, MHC-I^+^, and MHC-II^+^ cells than Alum-Pm or Pm (**Figures 5I–P**). The gating strategies for intracellular cytokine staining and pulmonary CD11b^+^ myeloid cell analysis are shown in **Supplementary Figure 1**. These results link TS-Pm immunization with increased cytokine-producing T-cell subsets and a more activated pulmonary CD11b^+^ myeloid phenotype before bacterial challenge.

### TS-Pm enhances protective efficacy after lethal *P. multocida* challenge

**Figure 6:**
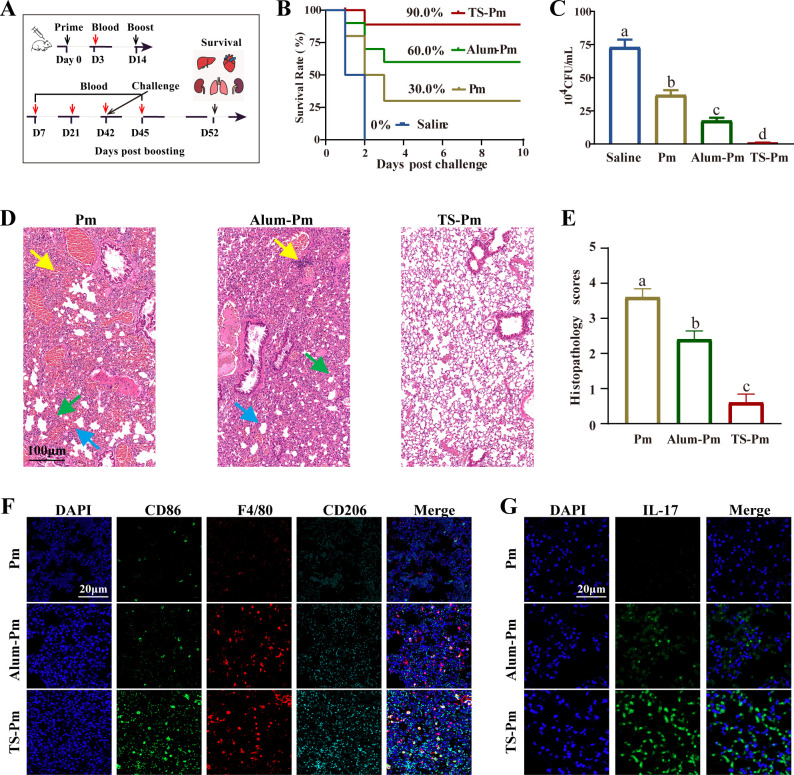
TS-Pm protects mice against lethal *Pasteurella multocida* challenge. **(A)** Immunization and challenge schedule. Mice were immunized twice with the indicated formulations and challenged intravenously with *P. multocida* (1 × 10^2^ CFU per mouse) on day 42 after booster immunization. **(B)** Survival after challenge (n = 10 mice per group; log rank test). **(C)** Terminal pulmonary bacterial burden in lungs collected from mice that died during the 10-day post-challenge observation period or from surviving mice euthanized at the scheduled day-10 endpoint. **(D)** Representative H&E stained lung sections at day 10 after challenge. Arrows indicate inflammatory infiltrates (yellow), hemorrhage or congestion (blue), and alveolar septal thickening or edema (green). **(E)** Lung pathology scores at day 10 after challenge (n = 5 mice per group). **(F, G)** Representative confocal immunofluorescence images of lung sections stained for F4/80, CD86, CD206 and IL-17. Nuclei were counterstained with DAPI. Images for each marker were acquired using identical settings across groups. Scale bar, 20 μm. Data in C and E are presented as mean ± SEM. Statistical significance was determined by one-way ANOVA followed by Tukey’s multiple comparisons test. Groups not sharing a letter differ significantly (P < 0.05). Additional lung immunofluorescence and multi organ histopathology are shown in **Supplementary Figure 3**.

Protective efficacy was evaluated using a lethal intravenous *P. multocida* challenge model (**Figure 6A**). After challenge, TS-Pm immunization improved survival to 90%, compared with 60% in the Alum-Pm group, 30% in the Pm group, and no survival in the saline group (**Figure 6B**). TS-Pm markedly reduced terminal pulmonary bacterial burden during the 10-day post-challenge observation period compared with the Saline, Pm, and Alum-Pm groups (**Figure 6C**).

Histopathological examination showed milder lung injury in the TS-Pm group, with less inflammatory infiltration, hemorrhage or congestion, and alveolar septal thickening than in the control groups (**Figure 6D**). Lung pathology scores were lower in the TS-Pm group than in the Alum-Pm and Pm groups (**Figure 6E**). Confocal immunofluorescence of lung sections showed less pronounced macrophage- and inflammation-related staining in TS-Pm-immunized mice after challenge (**Figures 6F, G**). Additional histological assessment of major organs and lung immunofluorescence staining is shown in **Supplementary Figure 3**. The survival, bacterial burden, histopathology, and immunofluorescence results collectively show that TS-Pm reduced disease severity after lethal *P. multocida* challenge.

### TS-Pm shows systemic tolerability in mice

Systemic tolerability was evaluated using hematological, antioxidant, biochemical, and histopathological parameters. Hematological analysis was performed at days 7, 21, and 42 after booster immunization and at day 45, corresponding to 3 days after challenge. TS-Pm induced changes in circulating leukocyte subsets consistent with immune activation, without hematological evidence of aggravated systemic toxicity (**Supplementary Figure 4**).

Antioxidant parameters were measured at day 21 after booster immunization and day 45 after challenge. Total antioxidant capacity, superoxide dismutase, glutathione peroxidase, and malondialdehyde did not indicate increased oxidative injury in the TS-Pm group compared with the control formulations (**Supplementary Figure 4**). Serum biochemical parameters were measured at day 3 after prime immunization and day 45 after challenge. ALT, AST, ALB, TP, CREA, and CK did not indicate increased hepatic, renal, or muscle injury after TS-Pm immunization and challenge (**Supplementary Figure 4**). Histological examination of major organs after challenge was also consistent with the absence of aggravated systemic tissue injury in the TS-Pm group (**Supplementary Figure 3**). These findings support the systemic tolerability of TS-Pm under the present immunization and challenge conditions.

### Draining lymph-node proteomics reveals immune remodeling after TS-Pm immunization

**Figure 7:**
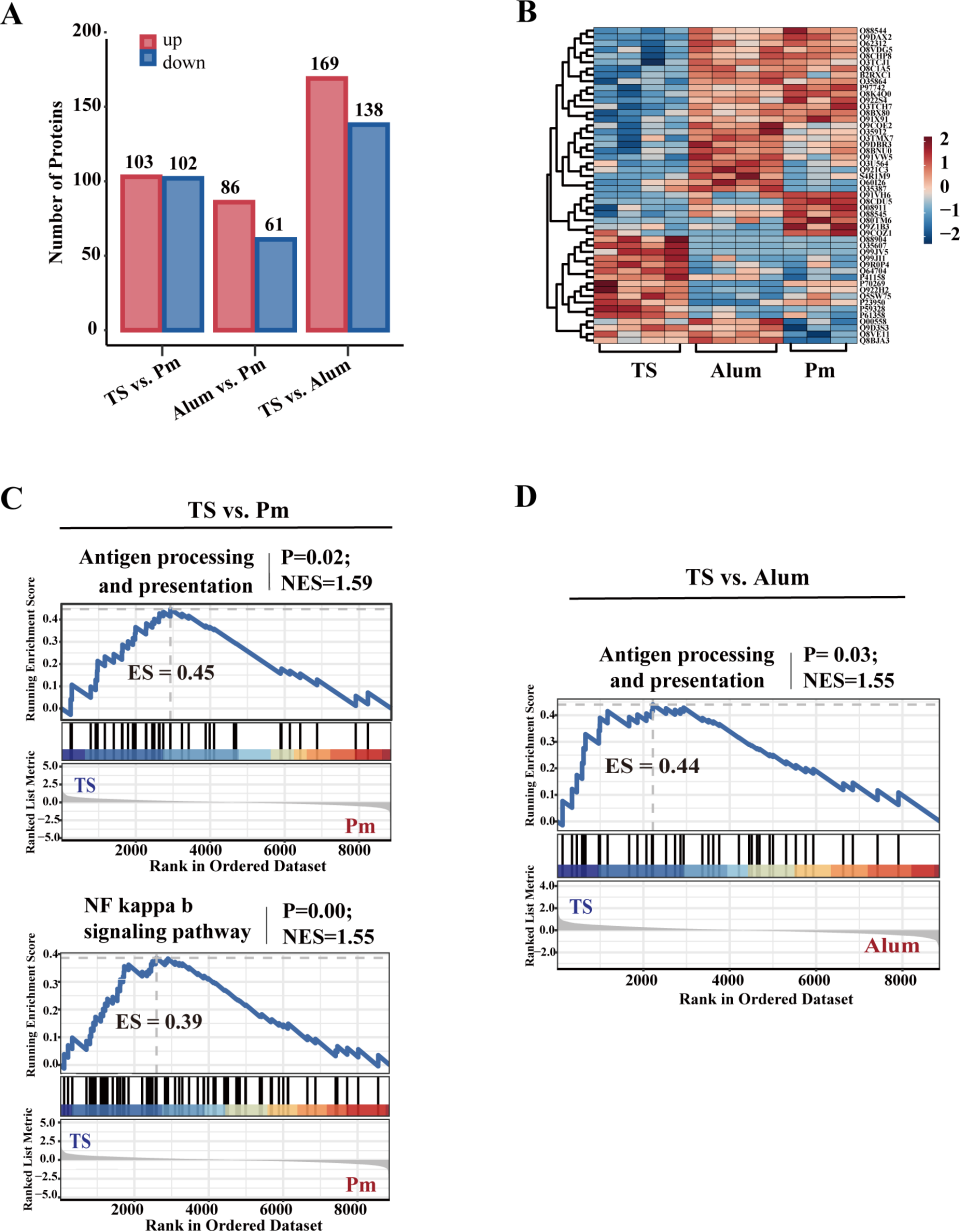
TS-Pm alters protein expression and immune pathway enrichment in draining lymph nodes. **(A)** Numbers of differentially expressed proteins identified in pairwise comparisons among Pm, Alum-Pm and TS-Pm groups. **(B)** Hierarchical clustering heatmap showing distinct protein abundance patterns among groups. **(C, D)** Gene set enrichment analysis of KEGG pathways for TS-Pm versus Pm and TS-Pm versus Alum-Pm comparisons. Draining lymph nodes were collected at day 21 after booster immunization. Data are based on n = 3 mice per group.

Draining lymph node proteomics was performed at day 21 after booster immunization to examine molecular changes associated with the immune response induced by TS-Pm. A total of 205, 147, and 307 differentially expressed proteins (DEPs) were identified in the TS-Pm versus Pm, Alum-Pm versus Pm, and TS-Pm versus Alum-Pm comparisons, respectively (**Figure 7A**). Hierarchical clustering separated TS-Pm from Alum-Pm and Pm based on draining lymph node protein abundance patterns (**Figure 7B**).

Gene set enrichment analysis showed enrichment of antigen processing and presentation and innate immune signaling pathways, including NF-κB signaling, in the TS-Pm group compared with Pm alone (**Figure 7C**). Antigen processing and presentation was also enriched in the TS-Pm versus Alum-Pm comparison (**Figure 7D**). Additional enrichment analyses showed changes in immune pathways involving antigen handling, inflammatory signaling, and immune-cell trafficking (**Supplementary Figure 5**). Protein interaction networks further highlighted immune modules containing proteins related to costimulation, antigen processing, inflammasome signaling, cytotoxicity, and chemokine trafficking, including CD40, TRAF6, TAP1, TAP2, NLRC4, PRF1, GZMB, and CXCR3 (**Supplementary Figures 6**–**S8**).

RT-qPCR was used to validate selected targets identified from the proteomic analysis. The mRNA expression trends of Nlrc4, Traf6, Tap1, Tap2, CD40, and Cxcr3 were generally consistent with the corresponding proteomic changes (**Supplementary Figure 9**). These molecular data were consistent with the cellular and protective outcomes and linked TS-Pm immunization to draining lymph node programs involving antigen processing and presentation, NF-κB-related signaling, and immune-cell trafficking.

## Discussion

Inactivated *P. multocida* vaccines are attractive for veterinary use because they are safe, easy to manufacture and antigenically broad, but weak cellular immunity and short-lived antibody responses remain important limitations. Recent studies have improved *P. multocida* bacterins and identified defined antigen candidates (19–21). Here, we examined whether a tea saponin–soybean oil submicron emulsion could improve the durability and protective efficacy of an inactivated *P. multocida* vaccine. TS-Pm induced more persistent antibody responses, stronger cellular immunity and greater protection than antigen alone or alum-adjuvanted vaccine. These findings indicate that submicron formulation of the whole-cell antigen affected not only response magnitude, but also the persistence and protective capacity of vaccine-induced immunity.

The formulation data distinguish TS-Pm from free antigen and Alum-Pm before immunological testing. Free inactivated *P. multocida* antigen showed marked aggregation, whereas TS-Pm remained well dispersed at approximately 300 nm and retained its morphology after storage. For inactivated whole-cell antigens, aggregation may reduce reproducible contact with phagocytic cells and contribute to variable immune activation. Particle size and antigen organization are recognized variables in adjuvant design (22, 23). Studies of oil-in-water nanoemulsions and related particulate adjuvants have further linked emulsion or particle properties with antigen uptake, macrophage activation, and vaccine efficacy (8–10). The smaller and more uniform structure of TS-Pm is therefore consistent with the stronger antigen uptake and macrophage activation observed *in vitro*. Compared with our previous vegetable-oil emulsions, which were larger and less uniform (17, 18), TS-Pm represents a more controlled plant-oil adjuvant formulation.

Tea saponin and soybean oil were selected for both immunological activity and practical veterinary use. Saponin-based adjuvants can enhance antigen presentation and cellular immunity, and studies of QS-21 and related saponin formulations have shown that this class of molecules can be strongly immunostimulatory (11, 12, 24). Soybean oil provides an accessible oil phase for emulsion preparation. In veterinary vaccines, adjuvant selection depends not only on immune potency, but also on tolerability, component availability, and field application. Conventional mineral-oil adjuvants are effective but may be limited by formulation-dependent reactogenicity and tissue persistence, whereas aluminum salts are safe and widely used but often provide limited stimulation of cellular immunity (4, 6). TS-Pm was designed as a plant-derived submicron emulsion that combines the immune-stimulatory activity of tea saponin with the formulation advantages of an oil-in-water system.

The macrophage experiments were guided by the respiratory tropism of *P. multocida* and the role of lung myeloid cells in bacterial defense. Lung macrophages participate in bacterial uptake, inflammatory regulation, and tissue repair during respiratory infection (25). Prior innate activation can also remodel monocyte-derived macrophage populations and improve antibacterial protection in the lung (26). Oil-in-water emulsion adjuvants interact early with monocytes and macrophage-lineage cells during antigen transport (27, 28). In our study, TS-Pm increased antigen uptake, inflammatory cytokine production, and costimulatory marker expression in RAW264.7 cells. In the CCK-8 assay, LPS was used only as a positive control, rather than as a dose-equivalent comparator with TS-Pm. *In vivo*, pulmonary CD11b^+^ myeloid cells displayed higher activation-associated markers before challenge. These findings support a relationship between TS-Pm formulation and phagocytic myeloid activation. Dendritic cells remain essential for vaccine priming, and their subset-specific role in the TS-Pm response was not resolved here. The macrophage-focused analysis was used because of its relevance to respiratory bacterial infection and to the pulmonary protection observed after challenge.

The antibody response induced by TS-Pm was sustained across the post-boost period. TS-Pm maintained higher Pm-specific IgG than Alum-Pm and increased both IgG1 and IgG2a. This response was therefore not limited to a transient increase after booster immunization or to a single antibody subclass. The increase in plasmablasts, plasma cells, and germinal center B cells provides a cellular basis for sustained antibody production. Germinal centers support B-cell selection, affinity maturation, and differentiation into memory and antibody-secreting cells (29), and sustained germinal center activity is closely linked to durable antibody responses after vaccination (30, 31). Antibody affinity, opsonophagocytic activity, and memory B-cell function were not measured in this study. Nevertheless, the antibody kinetics and B-cell subset data indicate that TS-Pm improved humoral persistence beyond an increase in peak antibody level.

TS-Pm also strengthened cellular immune responses. Increased *STAT4*, *STAT6*, and *RORγt* expression, together with higher IFN-γ, IL-4, IL-5, and IL-17, indicates that TS-Pm induced a mixed cytokine profile rather than a narrowly polarized response. IFN-γ can support macrophage antimicrobial activity (32), whereas IL-17 contributes to inflammatory cell recruitment and antibacterial defense at barrier tissues (33). Vaccine studies using combined innate stimuli have shown that Th1- and Th17-related responses can cooperate in protective immunity (34). The IL-4 and IL-5 responses suggest that helper activity supporting humoral immunity was also retained. This type of response may be useful for an inactivated extracellular bacterial vaccine, where antibody-mediated bacterial control and cellular support for phagocyte function are both likely to contribute to protection.

The protective effect of TS-Pm can be interpreted in relation to these humoral, cellular, and pulmonary myeloid responses. Mice receiving TS-Pm showed higher survival, lower pulmonary bacterial burden, and milder lung lesions after lethal *P. multocida* challenge. Protection against extracellular bacteria often involves antibody-dependent bacterial recognition, complement activation, and phagocyte-mediated clearance; complement pathways also interact closely with inflammatory and innate immune responses during infection (35). The sustained antibody response, cytokine-producing T cells, and activated pulmonary myeloid phenotype induced by TS-Pm are consistent with these protective mechanisms. The lung data also show a temporal distinction. Before challenge, TS-Pm increased activation markers on pulmonary CD11b^+^ myeloid cells; after challenge, TS-Pm-immunized mice showed less macrophage- and inflammation-related staining in lung sections. This pattern suggests that TS-Pm may prepare the lung myeloid compartment before exposure and reduce inflammatory tissue involvement once bacterial growth is controlled. For *P. multocida* infection, in which pathology reflects both bacterial replication and host inflammation, this balance is likely relevant to disease reduction.

Safety is an essential consideration for any adjuvant intended for veterinary use. Stronger adjuvant activity can be accompanied by reactogenicity, oxidative stress, or tissue injury. Under the conditions tested here, TS-Pm did not aggravate hematological toxicity, oxidative injury, serum biochemical indicators of liver, kidney, or muscle damage, or major-organ histopathology. Vaccine reactogenicity reflects inflammatory events that can limit acceptability even when immune responses are improved (36). Oxidative stress is also closely linked to inflammatory tissue damage and systemic stress responses (37). The current data support systemic tolerability in mice, while local injection-site reactions and target-animal safety remain important for future evaluation.

Draining lymph-node proteomics provided a molecular view of the immune environment induced by TS-Pm. Antigen processing and presentation were enriched in TS-Pm comparisons, and TAP1 and TAP2 appeared in the interaction network, consistent with MHC-related antigen handling (38). NF-κB-related signaling, NOD-like receptor-associated pathways, and proteins such as CD40, TRAF6, and NLRC4 were also represented, suggesting innate immune activation within draining lymph nodes (39, 40). Immune-cell trafficking was reflected by Cxcr3 validation, consistent with chemokine-dependent lymphocyte positioning (41). These pathway changes are in line with previous studies showing that saponin-based adjuvants are associated with lymphatic transport, antigen presentation, and adaptive immune responses, whereas oil-in-water emulsions can promote innate-cell recruitment and antigen transport to draining lymph nodes (7, 11, 24, 27, 28). Because the proteomic analysis used the complete TS-Pm formulation, these data cannot distinguish tea saponin-specific effects from soybean oil-related or combined effects. These molecular changes fit the cellular findings, including stronger B-cell differentiation and cytokine-producing T-cell responses. The proteomic results should be interpreted as an immune signature associated with TS-Pm immunization rather than evidence that individual pathways are required for protection.

Intravenous challenge was used because preliminary intranasal challenge attempts did not produce consistent mortality. This model allowed reproducible comparison of survival, bacterial burden, and lung pathology, but respiratory challenge models will be needed to assess airway protection more directly. The present work also emphasized macrophage-lineage responses and pulmonary CD11b^+^ myeloid cells, whereas dendritic-cell subsets and their contribution to T-cell priming were not defined. These points should be addressed in future studies using respiratory challenge models and more detailed analysis of antigen-presenting cell subsets.

In conclusion, TS-Pm improved the immune performance of an inactivated *P. multocida* vaccine by combining stable submicron antigen formulation with macrophage activation, persistent antibody responses, cellular immunity, reduced lung pathology, and draining lymph-node immune remodeling. The study extends our previous plant-oil adjuvant work from larger emulsions to a more uniform tea saponin–soybean oil submicron emulsion. These findings support plant-derived submicron emulsions as practical adjuvant candidates for veterinary vaccines that require durable humoral immunity, cellular immune activation, and protective efficacy across different antigen systems.

## Data Availability

The dataset has been deposited in the iProX repository under ProteomeXchange accession PXD071626.
