# Supplementary material for: Tea saponin–soybean oil submicron emulsion promotes durable protective immunity against Pasteurella multocida
Source: Front Immunol. 2026 Jul 20;17:1899202. doi: 10.3389/fimmu.2026.1899202 (PMC13429714; doi:10.3389/fimmu.2026.1899202)
Supplement: Supplementary file 1 [file DataSheet1.pdf]

Supplementary Material

Supplementary Figures

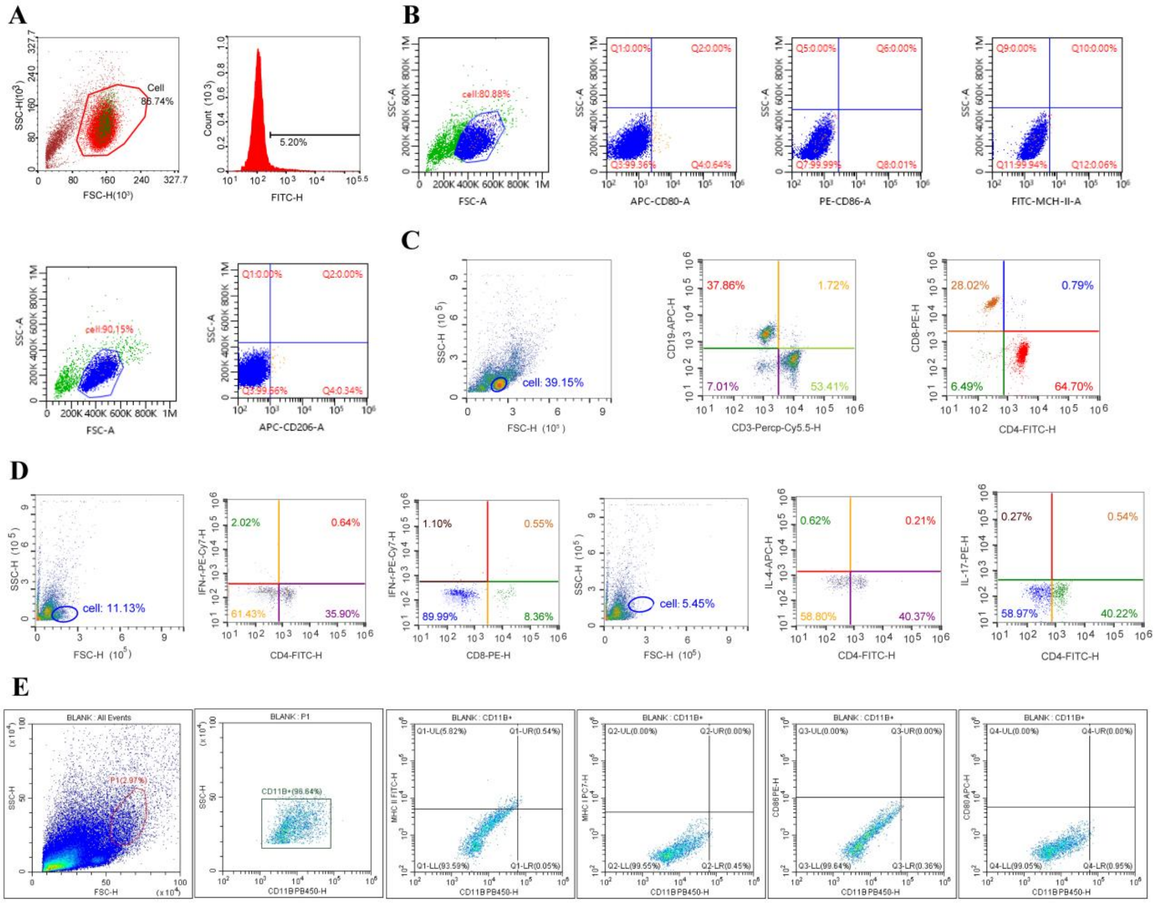

Supplementary Figure S1. Flow-cytometry gating strategies

(A) Gating strategy for FITC-OVA uptake by RAW264.7 cells. (B) Gating strategy for RAW264.7 macrophage surface phenotyping, including CD80, CD86, MHC-II, and CD206 expression. (C) Gating strategy for splenic lymphocyte subset analysis, including $CD3^{+}$ and $CD19^{+}$ populations and subsequent identification of $CD4^{+}$ and $CD8^{+}$ T-cell subsets. (D) Gating strategy for intracellular cytokine staining of splenic T cells, including $CD4^{+}IFN\text{-}\gamma^{+}$, $CD8^{+}IFN\text{-}\gamma^{+}$, $CD4^{+}IL\text{-}4^{+}$, and $CD4^{+}IL\text{-}17^{+}$ populations. (E) Gating strategy for pulmonary $CD11b^{+}$ myeloid-cell analysis, followed by assessment of CD80, CD86, MHC-I, and MHC-II expression.

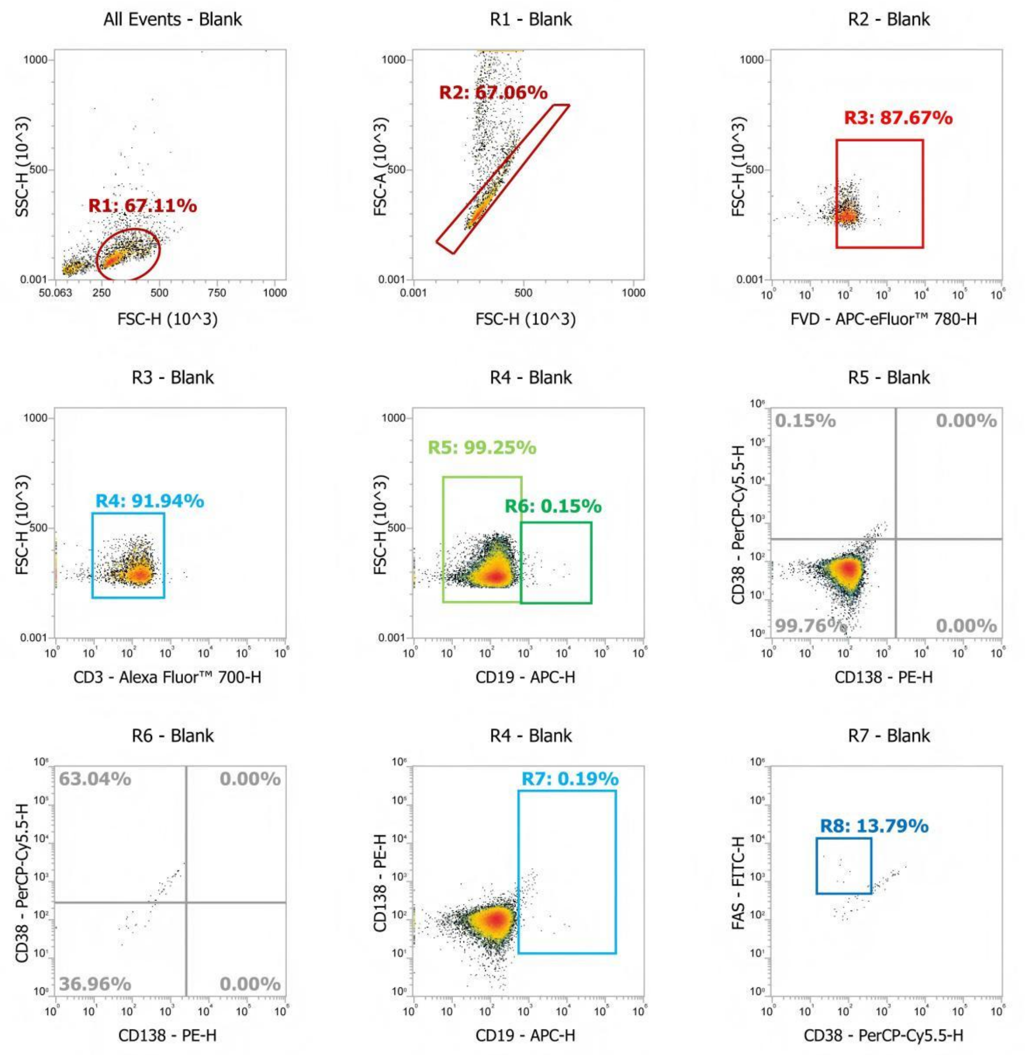

Supplementary Figure S2. Gating strategy for flow cytometric analysis of splenic B-cell subsets

Splenocytes were sequentially gated on the main cell population, singlets, live cells, and $CD3^{-}CD19^{+}$ B cells. Plasmablasts were identified as $CD19^{+}CD138^{-}CD38^{+}$ cells, plasma cells as $CD19^{+}CD138^{+}CD38^{+}$ cells, and germinal-center B cells as $CD19^{+}CD38^{+}Fas^{+}$ cells.

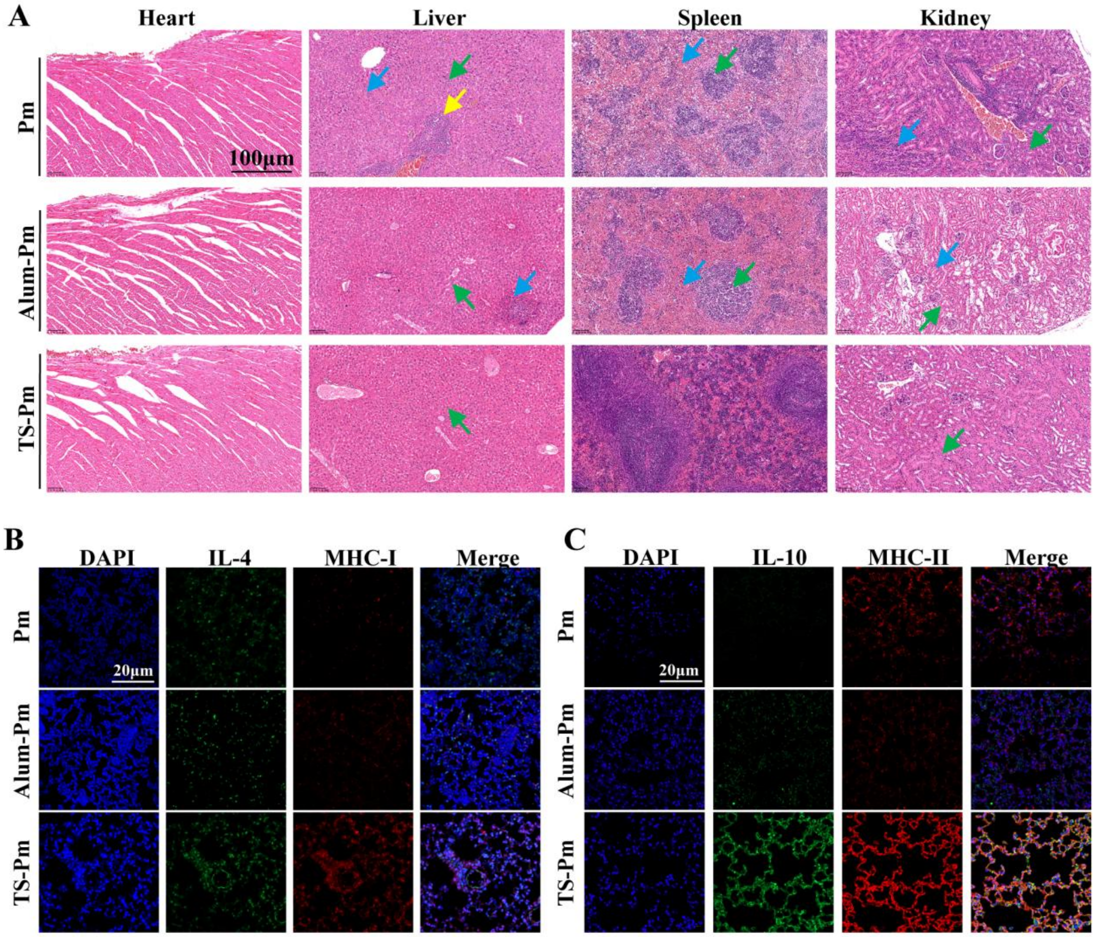

Supplementary Figure S3. Multi-organ histopathology and additional lung immunofluorescence after challenge

(A) Representative H&E staining of heart, liver, spleen, and kidney sections collected at day 10 post-challenge. Representative lesions are indicated by arrows: parenchymal cell swelling/degeneration (green), congestion (sinusoidal congestion in liver) (blue), and inflammatory cell infiltration (yellow) (scale bar, 100 μm). (B) Representative lung confocal immunofluorescence images stained for IL-4 and MHC-I (scale bar, 20 μm). (C) Representative lung confocal immunofluorescence images stained for IL-10 and MHC-II (scale bar, 20 μm).

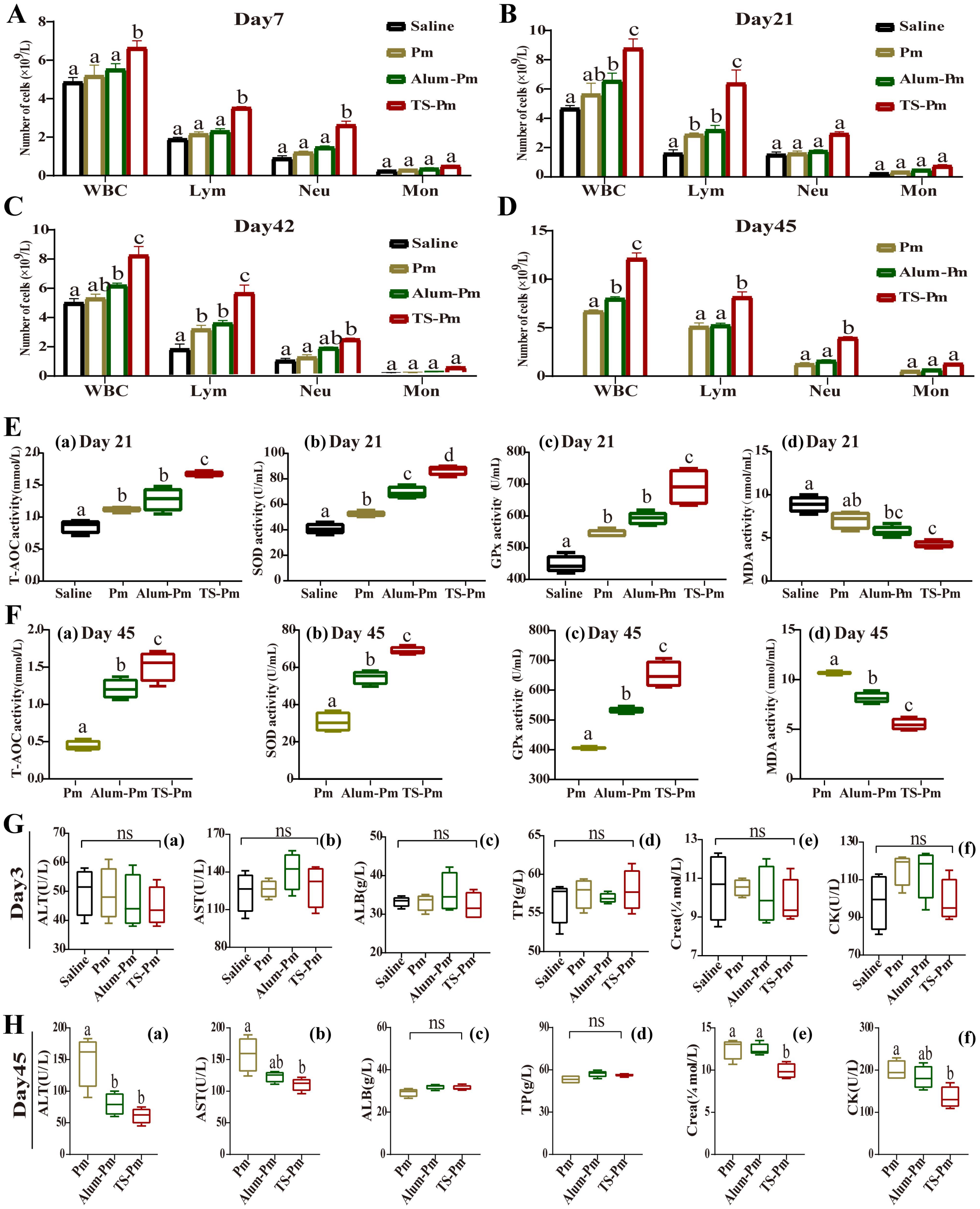

Supplementary Figure S4. Systemic tolerability assessments following TS-Pm immunization and challenge in mice

(A–D) Hematological parameters including total white blood cells (WBC), lymphocytes (Lym), neutrophils (Neu), and monocytes (Mon) measured at day 7, day 21, and day 42 after the booster immunization, and at day 45 (3 days post-challenge). (E–F) Antioxidant-related indices, including total antioxidant capacity (T-AOC), superoxide dismutase (SOD), glutathione peroxidase (GPx), and malondialdehyde (MDA), measured at day 21 and day 45 (3 days post-challenge). (G–H) Serum biochemistry indices, including ALT, AST, ALB, TP, CREA, and CK, measured at day 3 after the

prime immunization and at day 45 (3 days post-challenge). Data are presented as mean ± SEM (n = 6 mice per group). Groups not sharing a letter differ significantly (ANOVA with Tukey's multiple-comparisons test, $P < 0.05$); ns, not significant. For panels D, F, and H, no data were available for the Saline group at day 45 because all mice in this group died before the scheduled sampling time point after challenge.

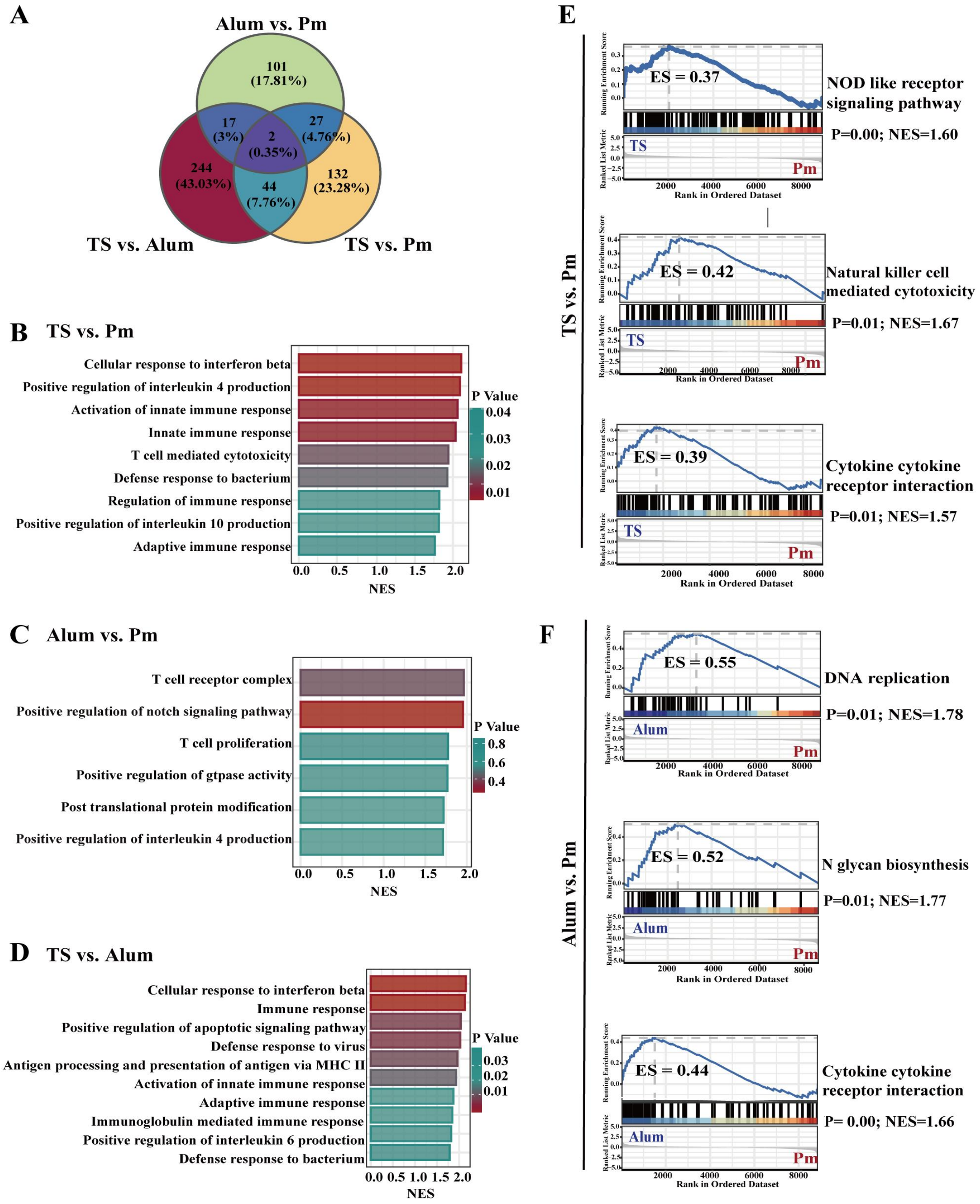

Supplementary Figure S5. Comprehensive enrichment analyses of draining lymph node proteomics

(A) Venn diagram showing the overlap of differentially expressed proteins among TS-Pm versus Pm (TS vs. Pm), Alum-Pm versus Pm (Alum vs. Pm), and TS-Pm versus Alum-Pm (TS vs. Alum) comparisons. (B–D) Functional enrichment analyses for TS vs. Pm (B), Alum vs. Pm (C), and TS vs. Alum (D). (E-F) Additional GSEA plots for TS vs. Pm and Alum vs. Pm.

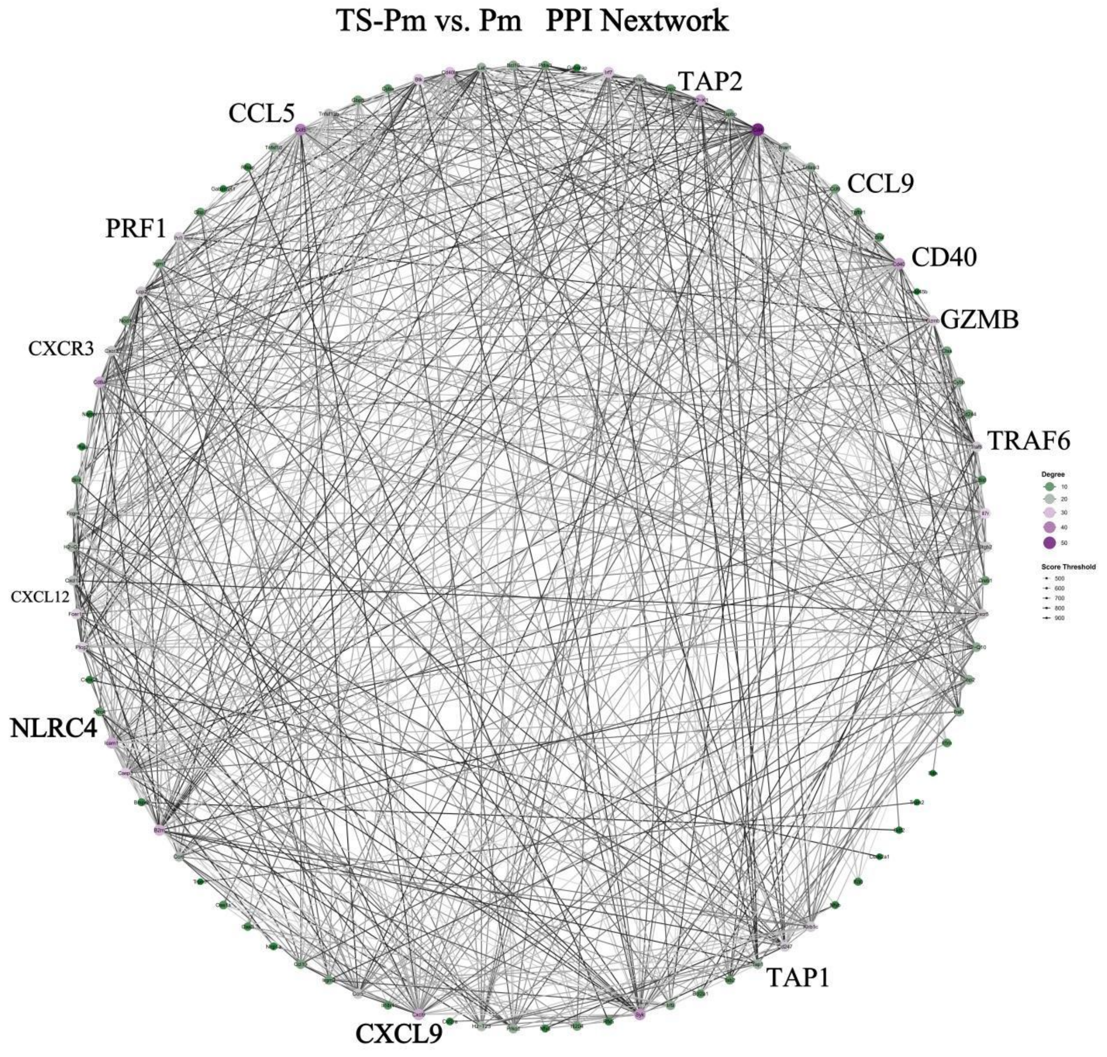

Supplementary Figure S6. Protein–protein interaction (PPI) network of differentially expressed proteins (DEPs) in TS-Pm vs. Pm.

The network was constructed using STRING and visualized in Cytoscape. Nodes represent differentially expressed proteins, and edges represent protein–protein associations based on STRING combined confidence scores.

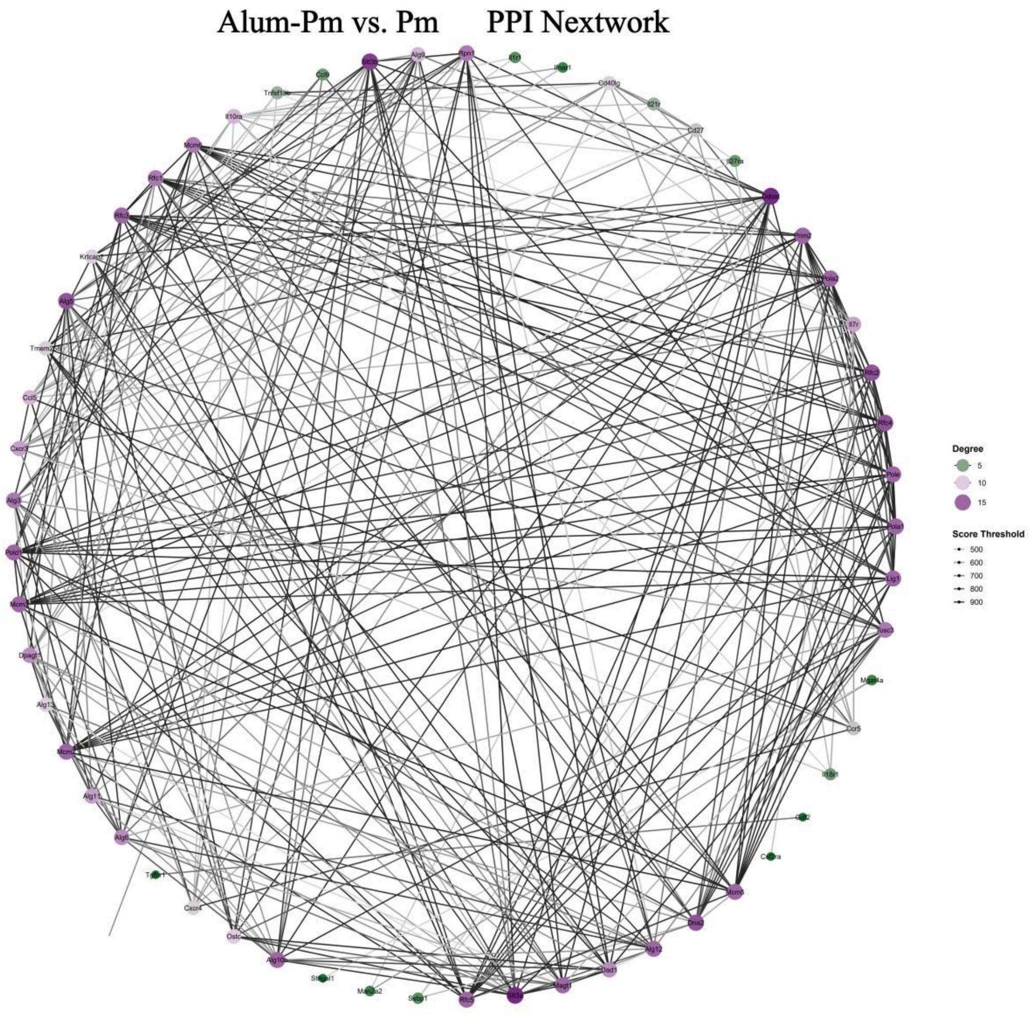

Supplementary Figure S7. Protein–protein interaction (PPI) network of differentially expressed proteins (DEPs) in Alum-Pm vs. Pm.

The network was constructed using STRING and visualized in Cytoscape. Nodes represent differentially expressed proteins, and edges represent protein–protein associations based on STRING combined confidence scores.

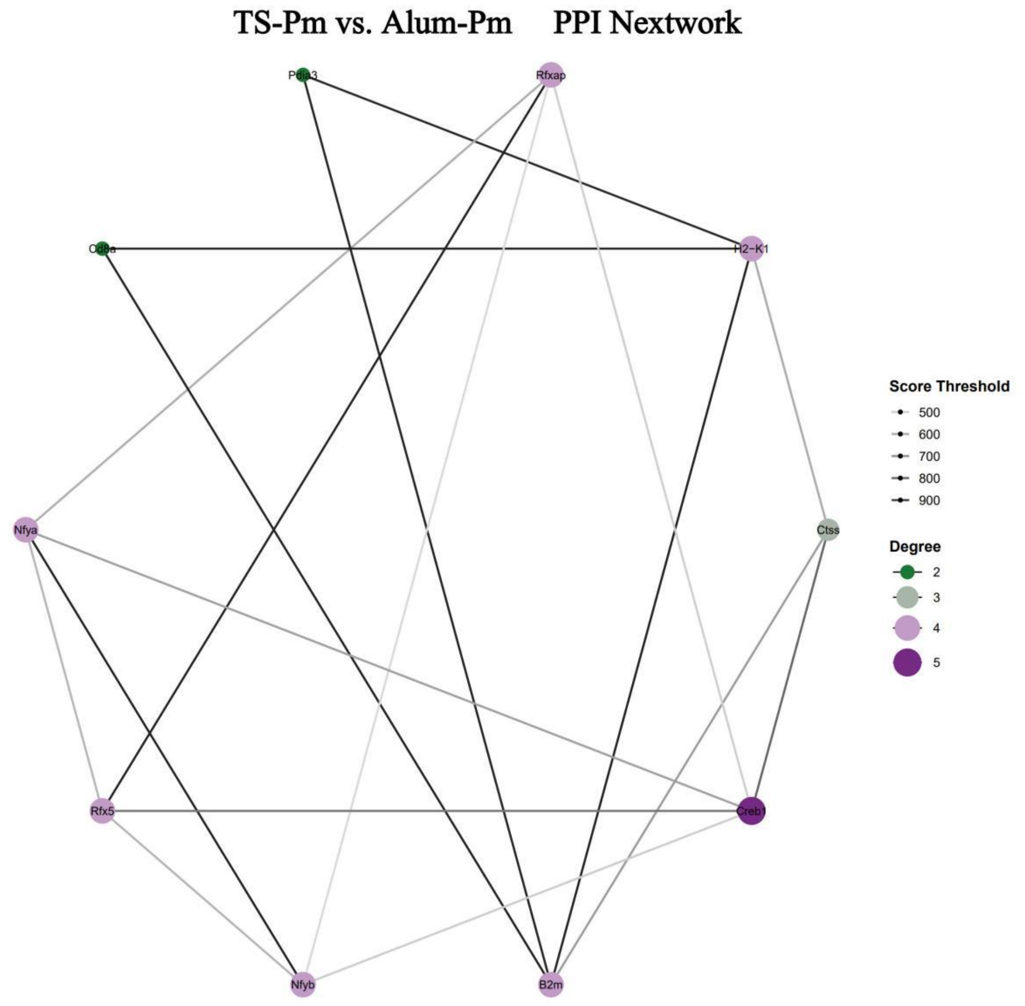

Supplementary Figure S8. Protein–protein interaction (PPI) network of differentially expressed proteins (DEPs) in TS-Pm vs. Alum-Pm.

The network was constructed using STRING and visualized in Cytoscape. Nodes represent differentially expressed proteins, and edges represent protein–protein associations based on STRING combined confidence scores.

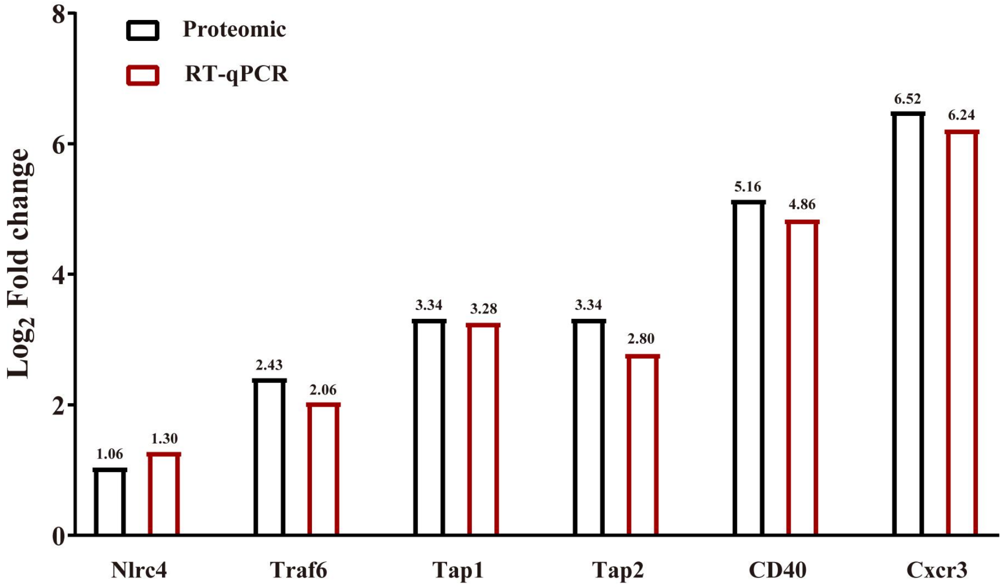

Supplementary Figure S9. RT-qPCR validation of selected genes identified from the proteomic analysis

Relative mRNA expression of *Nlrc4*, *Traf6*, *Tap1*, *Tap2*, *Cd40*, and *Cxcr3* was measured by RT-qPCR. Data are presented as $\log_2$ fold change relative to the Pm group and are shown as mean ± SEM (n = 3 mice per group).

Supplementary Table S1: Sequences of primers for quantitative RT-qPCR

Gene name	Primer sequence
(1) STAT4	Forward: CATCCCTGAAAACCCTCTGA<br>Reverse: GACATGGGGAGAAGGTCTG
(2) STAT6	Forward: CTCTGTGGGGCCTAATTTCCA<br>Reverse: CATCTGAACCGACCAGGAACT
(3) RORγt	Forward: CAGTATGTGGTGGAGTTTGC<br>Reverse: GCTTCCATTGCTCCTGCTTT
(4) Nlrc4	Forward: CTCACCACGGATGACGAACAGT<br>Reverse: TGTCATCCAGTATGAGTCTCTCG
(5) Traf6	Forward: TTTCCCTGACGGTAAAGTGCCC<br>Reverse: ACCTGGCACTTCTGGAAAGGAC
(6) Tap1	Forward: GACTCCTTGCTCTCCACTCAGT<br>Reverse: AACGCTGTCACCGTTCCAGGAT
(7) Tap2	Forward: AGCAGGAAGTCAGCCGCTACAA<br>Reverse: CGCAGTTCAGAATCAGCACCTG
(8) CD40	Forward: ACCAGCAAGGATTGCGAGGCAT<br>Reverse: GGATGACAGACGGTATCAGTGG
(9) Cxcr3	Forward: TACGATCAGCGCCTCAATGCCA<br>Reverse: AGCAGGAAACCAGCCACTAGCT
(10) GAPDH	Forward: TCGTCCGGTAGACAAAATGG<br>Reverse: GAGGTCAATGAAGGGGTCGT
